# Ethnic disparities in the longer-term clinical care and outcomes of stroke patients in England

**DOI:** 10.1371/journal.pone.0354353

**Published:** 2026-08-05

**Authors:** Eugene Yee Hing Tang, Uchenna Anyanwagu, Yu Fu, Christopher Price, The SSNAP Collaboration

**Affiliations:** 1 Stroke Research Group, Population Health Sciences Institute, Newcastle University, Newcastle upon Tyne, United Kingdom; 2 Department of Primary Care & Mental Health, Institute of Population Health, University of Liverpool, Liverpool, United Kingdom; 3 Sentinel Stroke National Audit Programme, Kings College London, London, United Kingdom; Cardiff University, UNITED KINGDOM OF GREAT BRITAIN AND NORTHERN IRELAND

## Abstract

Ethnic disparities in stroke incidence and recurrence are well-known but there is conflicting evidence regarding post-stroke care and recovery. In the UK, stroke-survivors are recommended a six-month specialist review but fewer than half receive this review, potentially exacerbating health inequities. Using data from the Sentinel Stroke National Audit Programme (SSNAP) from inception to August 2023 across four regions in England with varying ethnicity profiles we conducted a retrospective descriptive analysis to assess ethnic differences in six-month care and outcomes for stroke-survivors. A total of 80,413 (26.2%) stroke-survivors attended their 6-month review (whites: 26.5%, Black patients: 27.9%; Asian patients: 26.1%; Mixed patient: 27.6%; other: 23.5% other; unknown: 23.1%) with a higher proportion of reviews taking place via telephone for Black and Asian patients (X = 955.7, p = 0.001). After adjustment, non-White patients were significantly more likely to be followed up at 6 months (Black patients OR 1.29 (95%CI 1.21–1.38), Asian patients OR 1.10 (95% CI: 1.04–1.15). The odds of having a further stroke (Black OR 1.49 (95% CI: 1.21–1.84), Asian OR 1.25 (95% CI 1.03–1.52)) or other illness requiring hospital readmission (Black OR 1.25 (95% CI: 1.12–1.40), Asian OR 1.15 (1.05–1.27)) were significantly higher in Black and Asian patients following adjustment for age and stroke severity. At six months lipid therapy was significantly lower in Black patients (OR 0.79 (95% CI: 0.72–0.87)). Black and Asian stroke-survivors experience worse outcomes and lower use of secondary prevention despite similar clinic attendance. Culturally sensitive interventions are needed to reduce post-stroke complications.

## Introduction

Globally, the future incidence of stroke is likely to increase in both sexes, all age groups, and all socio-demographic groups [1]. Stroke also remains the second-leading cause of death and third-leading cause of death and disability [2]. Studies have shown higher incidence and death rates from stroke in Black patients compared to White patients. This is driven by the disproportionately higher incidences of hypertension and diabetes (which are known risk factors of stroke) in this group [3].

There are racial disparities in both stroke incidence [4] and stroke recurrence [5] in non-White ethnic patients. A higher proportion of White patients tend to present sooner following the onset of their stroke symptoms, receive thrombolysis and thrombectomy compared to minority ethnic patients in the USA [6]. It is yet unclear as to whether there are similar ethnic disparities in the post-stroke recovery period as there is a dearth of data on strategies to mitigate inequities in stroke rehabilitation [7]. In the UK, the healthcare system is free at the point of need. Current UK national guidance recommends that all stroke patients should receive a patient-centred structured review of their health and social care needs at 6 months after a stroke, usually in specialist settings, followed by an annual review in Primary Care [8]. Despite the importance in reviewing stroke patients particularly at the 6-month review, only around 40% of eligible patients actually receive this review [9]. There is a danger that not only are non-White individuals at heightened risk of stroke and the associated complications, there may also be further disparities in their subsequent post-stroke care. This also includes often hidden effects of stroke namely cognition. When compared to White stroke-survivors, Black patients are at increased risk of poorer neurocognitive outcomes [10]. Those from non-White (excluding white minorities) groups are also more likely to report worsening confusion or memory loss impacting their engagement in day-to-day activities [11]. As these are often the less visible deficits post-stroke, it is vital that services can detect and recognise these challenges to ensure earlier intervention and to reduce its impact particularly if there is a need to tailor this support to specific cultural groups.

The aim of this exploratory study was to describe aspects of post-stroke care and outcomes during the 6-month follow-up period and to assess whether there are any differences across non-White ethnic groups, and the impact of the COVID-19 lockdown.

## Materials and methods

### Data source

Data were on obtained from the Sentinel Stroke National Audit Programme (SSNAP) on the 30^th^ April 2024 following approval by the Healthcare Quality Improvement Partnership (HQIP). SSNAP is a national quality audit register used in collecting data on stroke care in all hospitals across England, Wales and Northern Ireland. Data are submitted by individual teams prospectively on patient demographics, clinical treatments and outcomes on all patients presenting with acute stroke from admission to 6 months after stroke (SSNAP - Home). Ethnicity was categorised as White, Black, Asian, Mixed and Others according to the clinical data inputted by the clinical team. As this was a retrospective analysis on clinical audit data, we anticipated that ethnicity can often be missing or not known, and these were defined according to the categories dictated by SSNAP. The authors did not have access to information that could identify individual participants during or after data collection.

### Study design and population

**Fig 1:**
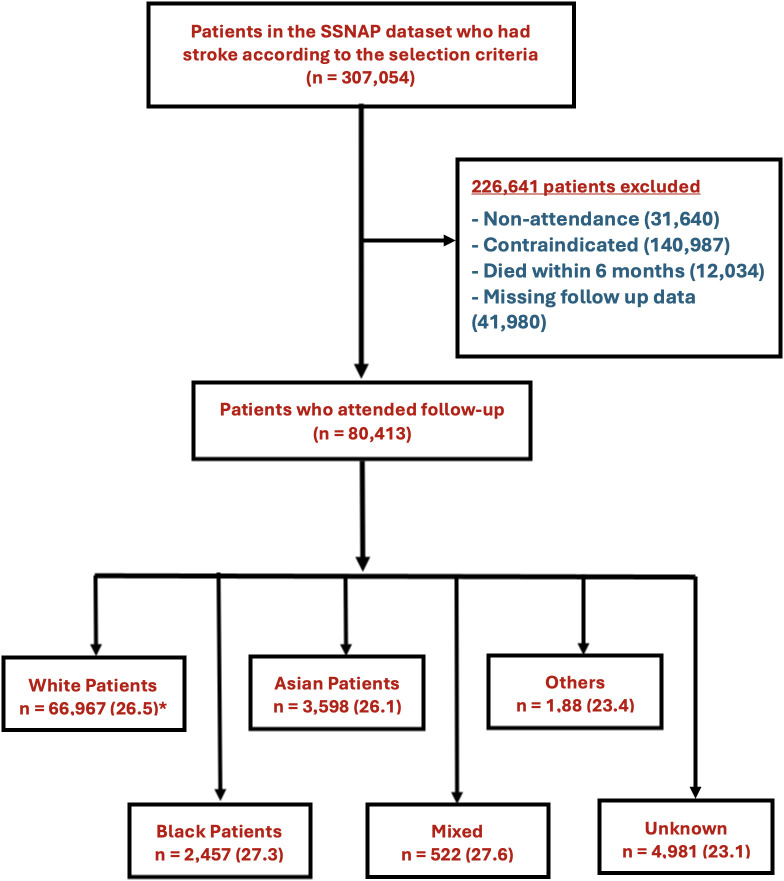
Participant summary flow chart. *The percentage represents the proportion of those who attended follow-up compared to the total number of patients in each ethnic category.

SSNAP is a prospective, registry-based cohort study of adult stroke patients aged ≥18 who were admitted to hospital across England, Wales and Northern Ireland. For this study, publicly available data from SSNAP were retrieved to order the 16 strategic clinical networks (SCN) by the proportion of Black and Asian stroke patients admitted. To ensure appropriate representation and mix of populations were included, data from four SCNs (London, East of England, South-East and North of England) were placed in order from the top to bottom quartiles for Black and Asian representation. Data were then requested from 4 SCNs, one from each quartile from SSNAP. The index date was defined as the date/time of onset/awareness of symptoms. All patients were eligible from inception to the 31st of August 2023. Data were retrieved from admission to their point of discharge from the registry, i.e., the six-month review. Fig 1 summaries how the study population was derived from the dataset.

### Statistical analysis

The study’s objectives were to describe the ethnic differences with regards to the 6 months follow-up period attendance, the impact of the COVID-19 lockdown on post-stroke on this, and the ethnic differences in post stroke care (clinical outcomes and use of medications).

Firstly, we stratified the entire study population by White vs Black vs Asian vs Mixed patients and others/unknown ethnicities, and used descriptive statistics to summarise the baseline demographics, comorbidities, use of medications, and stroke episode outcomes (S1 Table). Chi-square and T-test/linear regression analyses were used to compare differences between these among the ethnic groups.

To estimate the odds of follow up among the different ethnic groups, only data from those who attended the 6-month follow up were used (Fig 1). An exploratory analysis approach was used to examine the associations between the patients’ sociodemographic variables and the odds of follow up at 6 months within the ethnic groups First, we conducted a univariate logistic regression analysis to determine the variables that were associated with follow up at 6 months. Then all the significant variables in were fitted in the final multivariate model to adjust for their effect on the 6-month follow-up outcomes.

Secondly, to explore the impact that the COVID-19 lockdown had on the 6-month follow up, participants were divided into two cohorts – those whose stroke onset was before the 26th of March 2020 first lockdown versus after that. Follow up was categorised as binary (yes – attended or no – not attended). Patients who died before the follow up period, or in whom follow up was not indicated or with missing data were excluded. Pearson’s chi-square analysis was used to calculate the difference in the proportion between the two groups pre- and post-lockdown. Logistic regression model was fitted to explore the association between follow up within the ethnic groups in this period and this was adjusted for the lockdown period.

Finally, to explore the differences in post-stroke care (clinical outcomes and use of medications), the study population was stratified by their ethnicities and logistic regression model was fitted to explore the odds of each defined outcome in other ethnic groups compared to White patients. The univariate logistic regression models tested the association between each outcome the a priori baseline confounders. Only age and stroke severity (signified by the National Institutes of Health Stroke Scale (NHISS) score on arrival were associated with the outcomes. These were fitted in the final multivariate models to adjust for their confounding effect.

All point estimates were computed with 95% confidence intervals (CI), at the conventional statistical significance level of 0.05. Missing data in large proportion was classified as a different category for analysis. All data analyses were done using Stata Software, version 18.

### Ethical approval

SSNAP has permission granted under Section 251 approval by the Confidentiality Advisory Group of the Health Research Authority to collect the confidential patient information. The data retrieved following approval from the HQIP (Application number HQIP1450) provided non-identifiable patient data to the research team. The project also had ethical approval from Newcastle University’s Faculty of Medical Sciences (Reference: 35360/2023).

## Results

### Baseline characteristics

A total of 307,054 participants had a stroke within the defined period. Of this, 82.3% were White patients, while 2.9% Black patients, 4.5% Asian patients,0.6% mixed race patients, 2.6% other ethnic groups, and 7% unknown ethnicity (Fig 1).

As anticipated, the highest proportion of strokes was in the elderly with the age group 80–84 showing the highest proportion (16.2%) while this was 1.8% in those aged less than 40 (p < 0.001). There are slightly more males (51.9%) than females (48.1%). Asian patients had the highest proportion of patients with a modified Rankin Score (mRS) greater than zero compared to all other ethnic groups (55.1%, p < 0.001). More than a quarter of all the participants have had a previous stroke or TIA while a significant proportion had ischaemic stroke (87.3%) compared to other types (p < 0.001). S1 Table is the summary of the baseline demography, comorbidities, use of medication, and stroke episode outcomes of all the participants stratified by ethnicities.

### Six months review

**Fig 2:**
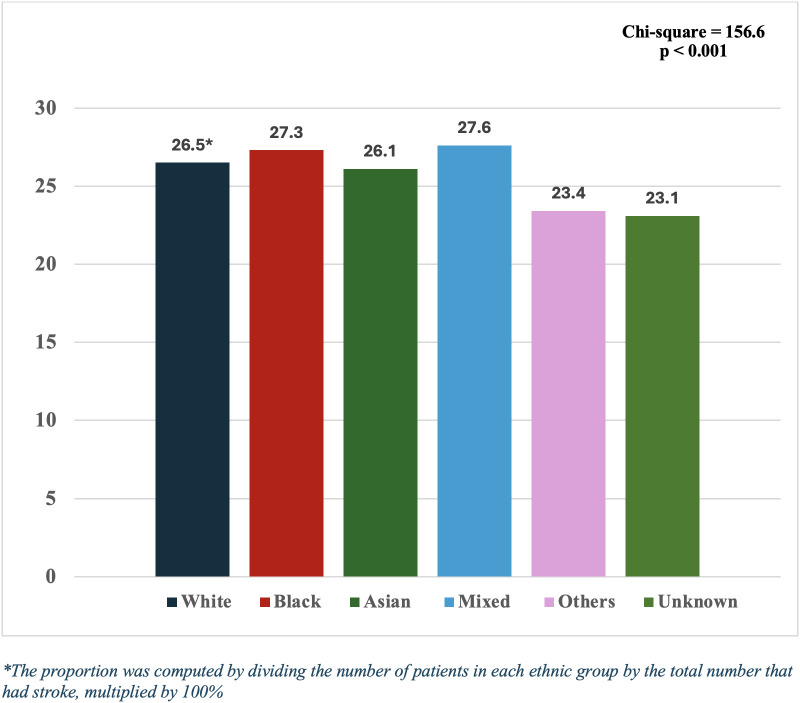
The proportion (%) of stroke survivors who attended their follow up at 6 months according to their ethnicities.

A total of 80,413 out of 307,054 (26.2%) participants attended their 6-month review. Of the total population in each ethnic group, 26.5% of White patients attended, 27.9% Black patients, 26.1% Asian patients, 27.6% Mixed patients, 23.5% other ethnicities, and 23.1% unknown (Fig 2). On the contrary, 10.3% defaulted for no reasons, 13.7% had missing records, 3.9% died within 6 months), while 46.0% were not able to be followed up because they either declined assessment or could not be contacted as they were not registered with a GP or have moved overseas, or had another stroke after being discharged from inpatient care and were readmitted into hospital.

Fifty-seven percent of the 6 months review was in-person, 42% via telephone, 0.48% online and 0.44% via post. More White patients, Mixed and the unknown groups were seen in person, while a greater proportion of Black patients, Asian patients, and other ethnic groups were reviewed via telephone (p = 0.001). A significant proportion of follow ups were carried out by a stroke coordinator. Compared to both the pre-stroke (1.0) and discharge mRS (2.0), the mRS at 6 months (2.2) was worse (higher) in all ethnic groups (p-value <0.001).

S2 Table outlines the baseline characteristics and clinical outcomes of patients who attended their six-month review by ethnicity. Those aged 75–84 years attended most compared to all other age groups across all ethnicities. Apart from the mixed ethnic group where equal proportion of males and females attended, more men attended follow up (p < 0.001). The proportion of participants with a pre-stroke mRS of zero was significantly greater in the unknown and mixed ethnic groups (54.5% each) than in others (51.8%), White patients (51.4%), Black patients (50%) and Asian patients (44.9%) ethnic groups (p < 0.001). The prevalence of heart failure was significantly lowest in the unknown ethnic group, while hypertension was significantly higher in Black patients (75%) compared to others (p < 0.001).

Compared to White patients, Black patients are 35% more likely have a follow up (OR 1.35, 95% CI 1.27–1.44) while Asian patients are 12% (OR 1.12, 95% CI 1.07–1.18), mixed 21% (OR 1.21 95% CI 1.06–1.38) and other ethnic groups 28% (OR = 1.28 95% CI = 1.19–1.38) more likely to attend. Conversely, the unknown ethnic group are 10% (OR = 0.90, 95% CI = 0.86–0.94) less likely to attend the 6-month follow up review compared to White patients. Following adjustment for age, gender, pre-stoke mRS, comorbidities (e.g., hypertension, atrial fibrillation, previous stroke/TIA and dementia), and the use of medications like antiplatelets and anticoagulants, these odds slightly reduced in all ethnic groups (Table 1)

**Table 1:** Odds and adjusted odds of follow up of stroke survivors at 6 months according to their ethnicities.

	Number (%)	Unadjusted			Adjusted*		
		OR	95% CI	p-value	OR	95% CI	p-value
White patients	66,967 (26.2)	1			1		
Black patients	2,457 (27.3)	1.35	1.27–1.44	<0.001	1.29	1.21–1.38	<0.001
Asian patients	3,598 (26.1)	1.12	1.07–1.18	<0.001	1.10	1.04–1.15	0.001
Mixed patients	522 (27.6)	1.21	1.06–1.38	0.004	1.17	1.03–1.34	0.020
Others	1,888 (23.5)	1.28	1.19–1.38	<0.001	1.22	1.13–1.32	<0.001
Unknown	4,981 (23.1)	0.90	0.86–0.94	<0.001	0.85	0.81–0.89	<0.001

**OR = Odds ratio. 95% CI = 95% confidence interval.**

*Adjusted for age, gender, mRS before stroke, hypertension, atrial fibrillation, previous stroke/TIA and dementia; and the use of antiplatelets and anticoagulants.

### 6-month follow-up review outcomes among the ethnic groups

The need for psychological support and the odds of change of residence of abode in all ethnic groups compared to White patients is shown in Table 2. After adjustment for age and stroke severity, the proportion of stroke-survivors being identified as requiring cognitive support was almost similar in Black and White patients, non-significantly lower in the mixed and other ethnic groups but was 19% in Asian patients and 24% higher in the unknown ethnic groups. The need for psychological support and the odds of change of residence of abode in all ethnic groups compared to White patients are summarised in Table 2.

**Table 2:** Odds ratio showing differences in important clinical outcomes in stroke survivors who attended their six months follow up reviews according to their ethnicities.

Ethnicity	Number (%)	Cognitive support	Psychological support	Change in residence	Atrial fibrillation	Myocardial Infarction	Another stroke	Hospital readmission
		OR (95%CI)	OR (95%CI)	OR (95%CI)	OR (95%CI)	OR (95%CI)	OR (95%CI)	OR (95%CI)
**White patients**	66,967 (26.2)	1 (reference)	1 (reference)	1 (reference)	1 (reference)	1 (reference)	1 (reference)	1 (reference)
**Black patients**	2,457 (27.3)	0.99 (0.89-1.09)	1.31 (1.14-1.50)	1.19 (1.03-1.38)	0.63 (0.56-071)	0.88 (0.48-1.61)	1.49 (1.20-1.84)	1.25 (1.12-1.40)
**Asian patients**	3,598 (26.1)	1.19 (1.08-1.30)	1.53 (1.35-1.74)	0.57 (0.49-0.61)	0.55 (0.50-0.61)	1.86 (1.30-2.66)	1.25 (1.03-1.52)	1.15 (1.05-1.27)
**Mixed patients**	522 (27.6)	0.94 (0.75-1.17)	0.99 (0.76-1.31)	1.05 (0.76-1.46)	0.66 (0.51-0.85)	0.38 (0.05-2.71)	1.11 (0.66-1.86)	0.87 (0.67-1.14)
**Others**	1,888 (23.5)	0.91 (0.81-1.03)	1.22 (1.04-1.43)	0.70 (0.58-0.86)	0.73 (0.64-0.83)	1.14 (0.62-2.09)	1.48 (1.16-1.89)	0.92 (0.80-1.05)
**Unknown**	4,981 (23.1)	1.24 (1.14-1.34)	1.39 (1.25-1.55)	0.75 (0.70-0.84)	0.80 (0.75-0.87)	0.78 (0.50-1.23)	0.77 (0.63-0.94)	0.81 (0.74-0.89)

OR = Odd Ratio adjusted for age and stroke severity*. 95%CI = 95% confidence interval.

(The National Institutes of Health Stroke Scale (NHISS) score on arrival was used as a marker of stroke severity.

Table 2 also summarises the odds of relevant clinical comorbidities like atrial fibrillation, myocardial infarction, the incidence of another stroke/TIA and the risk of hospital readmission in all other ethnic groups compared to White patients.

The odds of the use of secondary prevention medications are summarised in Table 3. Lipid therapy use was significantly lower in Blacks (21%), mixed (24%) and others (21%) ethnic groups but non-significantly higher in Asian patients (5%) (Table 3). Conversely, antihypertensive use was significantly higher in non-White ethnicities.

**Table 3:** Odds ratio showing differences in the use of medications in stroke survivors who attended their six months follow up reviews according to their ethnicities.

Ethnicity	Number (%)	Antihypertensives	Lipid therapy
		OR (95%CI)	OR (95%CI)
White patients	66,967 (26.2)	1 (reference)	1 (reference)
Black patients	2,457 (27.3)	1.98 (1.79-2.20)	0.79 (0.72-0.87)
Asian patients	3,598 (26.1)	1.52 (1.40-1.65)	1.05 (0.96-1.14)
Mixed patients	522 (27.6)	1.45 (1.18-1.77)	0.76 (0.63-0.93)
Others	1,888 (23.5)	1.17 (1.06-1.30)	0.79 (0.71-0.88)
Unknown	4,981 (23.1)	1.04 (0.97-1.10)	0.85 (0.79-0.91)

### Impact of COVID- 19 on the 6-month follow up among the ethnic groups

Compared to the pre-COVID-19 lockdown period, a significant proportion of 6-month follow up occurred after the COVID-19 lockdown (67% vs 53%, p < 0.001), Odds ratio = 1.65, 95% CI: 1.62–1.69, p < 0.001.

Following adjustment for the lockdown period, Black patients were 29% (OR = 1.29, 95% CI = 1.21–1.37, p < 0.001) more likely to attend their follow up compared to White patients. In Asian patients, this was 9% (OR = 1.09, 95% CI = 1.04–1.15, p = 0.001), 18% in the mixed ethnic group (OR = 1.18, 95% CI = 1.04–1.35, p = 0.013), and 24% in others (OR = 1.24, 95% CI = 1.16–1.34, p < 0.001). However, in the unknown ethnic group category, the odds of attending follow up was 12% lower (OR = 0.88, 95% CI = 0.84–0.92, p < 0.001) compared to White patients.

## Discussion

Our findings demonstrate despite non-White stroke survivors having the highest attendance rate for 6-month follow-up, there were disparities in health outcomes. This did not change even when accounting for the COVID-19 period. Post-stroke comorbidities were more likely in all other ethnic groups combined, in particular recurrent stroke, and hospital readmissions for other illnesses in Black and Asian stroke-survivors. The odds of having a myocardial infarction within six months post-stroke was also significantly higher in Asian patients. Conversely, atrial fibrillation pre- and post-stroke was less likely in non-White stroke-survivors. There were differences in use of secondary prevention. Black and Asian stroke-survivors were more likely to be taking antihypertensives, which may reflect the fact that a higher proportion had hypertension pre-stroke. However, all other ethnic groups combined stroke survivors are less likely to be taking lipid therapy. There was a similar level of identification of need for cognitive support in Black and Mixed stroke-survivors compared to White patients with Asian patients identified as needing more psychological support.

The UK population is becoming increasingly more ethnically diverse with the 2021 UK census identified that the majority of the population remained Caucasian patients (81.7%), followed by Asian patients (9.3%), Black patients (4.0%), Mixed ethnicity patients (2.9%) and others (2.1%)(1). To capture a generalisable sample of stroke-survivors we requested a representative dataset from SSNAP with one SCN from each quartile by the proportion of those from Black and Asian ethnicities. This resulted in a generalisable sample as our study is roughly representative of the general population with most of the stroke-survivors being White patients (82.3%), followed by Asian patients (4.5%), Black patients (2.9%), Mixed ethnicity patients (0.6%) and other (2.6%) ethnic groups with 7% of unknown ethnicity. Importantly the proportion of all other ethnic groups combined patients in the UK has increased between 2011–2021 across all ethnicities [12]. It is therefore important primary, and specialist clinical services find ways to address any inequities in healthcare outcomes.

Attendance to the 6-months follow up review is generally low (26.2%), which is slightly lower than national data [13]. Although non-White patients were more likely to attend their 6-month review, a greater proportion of Black patients, Asian patients, and other ethnic groups were reviewed via telephone rather than in person. For those who attended their 6-month reviews, non-White patients were more likely to have comorbidities at baseline including hypertension and diabetes. This may be a factor in the higher recurrent stroke rate. Previous studies have found that Black patients, particularly at younger ages, were more strongly linked with both earlier acute onset [14] and recurrence [15] and may not be fully attenuated by traditional stroke risk factor management [5]. This difference was not confined to recurrent stroke as both hospitalisation for other illnesses and myocardial infarction at 6-month follow-up were higher in all other ethnic groups combined patients in our study. This suggests that there may be benefits to earlier or more targeted follow-up for these at-risk populations. Non-attenders for 6-month reviews tended to be younger in non-White patients (75–85 years age group among White patients, 55–64 years in Black patients; 65–69 years in Asian patients and 60–64 years mixed ethnic group), and were more likely to have risk factors for recurrent stroke such as hypertension in other than White stroke-survivors would appear to be a group that should be particularly targeted. Previous studies comparing immigrant health versus long-term residents, immigrants (mainly from Asian ethnicities) had a higher risk of being disabled following a stroke on discharge compared to long-term residents in a comparable healthcare system to the UK [16]. Future research should look at where in the stroke care pathway would be best to intervene to reduce the impact of stroke for those from minority ethnic populations.

Although racial and ethnic disparities are well known and persistent, less is known around how health services should be tackling these inequalities. The impact of race on post-stroke outcomes is just one of the social determinants of health which drives health inequalities. Those from Asian ethnicities have increased odds of undiagnosed hypertension and diabetes [17] which in turn increases the risk of stroke. Upon acute presentation of non-White populations are less likely to use emergency medical services and then tend to receive lower rates of treatment with thrombolysis or mechanical thrombectomy [6]. Factors such as cultural beliefs, lack of knowledge around stroke, language barriers and trust in clinical services may explain some of these disparities [7]. Delay in treatment could ultimately lead to worsening post-stroke outcomes and acute services also need to be mindful of these ethnic disparities. To address this delayed presentation, the Stroke Warning Information and Faster Treatment (SWIFT) trial compared the effect of an interaction intervention with enhanced educational materials on recurrent stroke arrival times in a multiethnic prospective cohort of both stroke and transient ischaemic attack survivors [18]. The SWIFT educational arms resulted in a significant increase in the proportion of patients arriving within <3 hours from symptom onset in both the intensive intervention group and those receiving enhanced usual care. There was no detectable difference between the two groups [19] which may be because the comparator arm was enhanced usual care. The comparison to enhanced usual care is not necessarily reflective of real-world clinical practice particularly given that different healthcare services can potentially offer heterogeneous packages of care post-stroke depending on locality.

In our UK sample, secondary prevention in the form of lipid therapy was found to be reduced particularly in Black patients, Mixed ethnicity patients, or other ethnicities. In Canada, which is a similar healthcare system to the UK, immigrants were more likely to achieve cholesterol targets [20]. Good lipid control is a mainstay of post-stroke management not only to reduce recurrent stroke but may also reduce adverse cognitive deficits such as dementia, which is known to be increased post-stroke [21,22]. Most randomized controlled trials which have tested the effectiveness of culturally-tailored interventions have tended to use blood pressure reduction as an outcome [7]. The Systemic Use of Stroke Averting Interventions (SUSTAIN) trial, which was conducted in a multi-racial/multi-ethnic vulnerable population, tested a chronic care model-based intervention consisting of group and individual clinics, self-management support, report cards and decision support [23]. Helpfully, African Americans not only had improved systolic blood pressure in the intervention arm, but there were also improvements in the lowering of low-density lipoprotein [23]. Working with underserved communities to understand their needs and their barriers to secondary prevention medication adherence is key.

Cognitive impairment is common post-stroke and also associated with ethnicity [24]. Neuropsychiatric outcomes such as depression, anxiety, fatigue, and apathy are common post-stroke [25]. Depression post-stroke has an estimated pooled prevalence of around a third (27%) from observational studies. Those experiencing depression within 3 months post-stroke more at risk of remaining depressed a year after stroke [26]. Similarly post-stroke anxiety can occur for 1 in 4 stroke-survivors [27]. Psychological outcomes such as depression and anxiety are often linked to cognitive deficits. Links between ethnicity and post-stroke outcomes such as depression are not consistent [28] with some finding no differences [29]. There do seem to be some ethnic differences when it comes to cognition with more cognitive impairment suggestive of dementia, found in non-Europeans compared with Europeans [30]. Post-stroke cognitive impairment is more common in those from Caribbean/African ethnicities [24]. In our study, at follow up, the use of psychological support was higher in Asian patients compared to White patients. Conversely, those from Mixed or Black ethnicities had similar levels to Whites in terms of receiving cognitive support. It is known that post-acute access to clinical psychology is often limited and below recommendations [31]. The degree of utilization of cognitive support offered to Black patients may be more of a reflection of the available clinical services or to cultural understanding of what normal or abnormal cognition may entail. Clinical services need to find ways to not only identify these individuals requiring psychological and/or cognitive support but also ways of following them up to ensure earlier detection of common post-stroke outcomes such as depression, anxiety, and cognitive impairment.

Although our exploratory has a number of strengths such as being representative of the national ethnicity profile, it has some inherent limitations. As a routinely collected audit across the 4 nations of UK, it runs a major risk of incomplete data entry by the clinical teams due to its prospective nature, and missing data especially relating to the ethnicities. Having up to 7% unknown and 2.6% ‘Others’ ethnic groups could potentially introduce a non-differential bias to our findings. The low 6-month follow-up data could be indicative of a poor reflection of the post-acute stroke population. Therefore, our findings should be interpreted with caution. SSNAP is a clinical prospective audit of all stroke survivors whose input is reliant on the clinical teams looking after the stroke patient. Whilst there is a high proportion, we have deemed missing due to lack of follow-up data, this is mainly since stroke-survivors have not attended or have no record of attendance. There will of course be clinical reasons for their non-attendance. This is reflected nationally where the proportion of patients receiving a 6 month stroke review is consistently only just over a third of all eligible stroke patients [32]. Techniques to address missing data, e.g., multiple imputation were considered but there were concerns that this would only serve to increase bias in the results as there would not be enough justification that the data were missing at random [33]. This was an issue when conducting the analysis around the odds of the patient taking their antiplatelets and anticoagulant therapy as there was significant missingness in the baseline data, which meant meaningful conclusions around this aspect would be biased by the missingness of the baseline data. Further, given the size of the dataset it would seem unlikely that spurious associations are being reported (especially if these show effects in a similar direction). We were therefore interested in those who do manage to attend their reviews and this exploratory study highlights areas for future research and implications for clinical teams to consider. Finally, we analysed all stroke-survivors together irrespective of underlying cause. As this is an exploratory study we wanted to assess the clinical outcomes of all stroke-survivors and to look at the standards of care across ethnicities rather than any mechanistic causes. Further, in routine clinical services in the UK, all stroke-survivors receive the same follow-up care post-stroke. We do recognise that there may be some underlying associations between type of stroke and ethnicity which could confound our interpretations and future studies could look more specifically on how subtype of stroke could influence post-stroke care.

## Conclusions

Our exploratory study using real-world clinical audit data provides novel insights into the ethnic differences in six-month outcomes following stroke. Patients from all other ethnic groups combined may get the opportunities for routine specialist follow-up but prior to this 6-month review, they have already had poorer health outcomes post-stroke including myocardial infarction, recurrent stroke, and hospitalisation for other illnesses. Our findings have important clinical implications to emphasise the need for community and primary care services to support these individuals during the earlier post-stroke period in partnership with specialist services. Future research is needed to understand the reasons behind these ethnic differences in post-stroke outcomes and to work together with these at-risk communities to developed culturally sensitive interventions to address these disparities in post-stroke outcomes.

## Supporting information

S1 Table Baseline demography, comorbidities, use of medication, and stroke episode outcomes of all the stroke survivors stratified by their ethnicities. (DOCX)

S2 Table Baseline Characteristics and Clinical Outcomes in only stroke survivors who attended their 6 months follow up according to their ethnicities. (DOCX)
