# Supplementary material for: Ethnic disparities in the longer-term clinical care and outcomes of stroke patients in England
Source: PLoS One. 2026 Aug 5;21(8):e0354353. doi: 10.1371/journal.pone.0354353 (PMC13440867; doi:10.1371/journal.pone.0354353)
Supplement: Supplementary file 1 [file pone.0354353.s001.docx]

# **Supporting Information**

**S1 Table. Baseline demography, comorbidities, use of medication, and stroke episode outcomes of all the stroke survivors stratified by their ethnicities.**

| **Baseline Variable** | **White**  **N (%) * = 252,749 (82.3)** | **Black**  **N (%) = 9,003 (2.9)** | **Asian**  **N (%) = 13,806 (4.5)** | **Mixed**  **N (%) = 1,895 (0.6)** | **Others**  **N (%) = 8,050 (2.6)** | **Unknown**  **N (%) = 21,551 (7.0)** | **Total Number**  **N (%) = 307,054 (100)** | **P value** |
| --- | --- | --- | --- | --- | --- | --- | --- | --- |
| **Demographics. N (%)** |  |  |  |  |  |  |  |  |
| **Age categories** |  |  |  |  |  |  |  |  |
| <40yrs | 3,618 (1.4) | 334 (3.7) | 522 (3.8) | 98 (5.2) | 331 (4.1) | 516 (2.4) | 5,419 (1.8) |  |
| 40-44yrs | 2,917 (1.2) | 339 (3.8) | 471 (3.4) | 74 (3.9) | 246 (3.1) | 463 (2.2) | 4,510 (1.4) |  |
| 45-49yrs | 5,466 (2.2) | 575 (6.4) | 635 (4.6) | 113 (6.0) | 395 (4.9) | 769 (3.6) | 7,953 (2.6) |  |
| 50-54yrs | 9,287 (3.7) | 887 (9.9) | 875 (6.3) | 153 (8.1) | 584 (7.3) | 1,185 (5) | 12,971 (4.2) |  |
| 55-59yrs | 12,887 (5.1) | 1,059 (11.8) | 1,086 (7.9) | 161 (8.5) | 724 (9.0) | 1,493 (6.9) | 17,410 (5.7) |  |
| 60-64yrs | 16,809 (6.7) | 1,037 (11.5) | 1,520 (11.1) | 197 (10.4) | 830 (10.3) | 1,817 (8.4) | 22,210 (7.2) |  |
| 65-69yrs | 22,198 (8.8) | 849 (9.4) | 1,668 (12.1) | 149 (7.9) | 790 (9.8) | 2,137 (9.9) | 27,791 (9.1) | <0.001 |
| 70-74yrs | 30,669 (12.1) | 823 (9.1) | 1,601 (11.7) | 222 (11.7) | 900 (11.2) | 2,618 (12.2) | 36,833 (12.0) |  |
| 75-79yrs | 37,882 (15.0) | 1,027 (11.4) | 1,822 (13.2) | 226 (11.9) | 979 (12.2) | 2,897 (13.4) | 44,833 (14.6) |  |
| 80-84yrs | 42,703 (16.9) | 1,048 (11.6) | 1,790 (13.0) | 228 (12.0) | 981 (12.2) | 3,047 (14.1) | 49,797 (16.2) |  |
| 85-89yrs | 38,584 (15.3) | 648 (7.2) | 1,214 (8.8) | 187 (9.9) | 751 (9.3) | 2,642 (12.3) | 44,026 (14.3) |  |
| 90+above | 29,729 (11.8) | 377 (4.2) | 602 (4.4) | 87 (5.6) | 539 (6.7) | 1,967 (9.1) | 33,301 (10.9) |  |
|  |  |  |  |  |  |  |  |  |
| **Gender** |  |  |  |  |  |  |  |  |
| Male | 129,235 (51.1) | 4,751 (52.8) | 8,079 (58.5) | 996 (52.6) | 4,524(56.2) | 11,670 (54.2) | 159,255 (51.9) | <0.001 |
| Female | 123,514 (48.9) | 4,252 (47.2) | 5,727 (41.5) | 899 (47.4) | 3,526(43.8) | 9,881 (45.9) | 147,799 (48.1) |  |
|  |  |  |  |  |  |  |  |  |
| **Employment Status** |  |  |  |  |  |  |  |  |
| Full time | 2,754 (1.1) | 288 (3.2) | 255 (1.9) | 45 (2.4) | 168 (2.1) | 407 (1.9) | 3,917 (1.3) |  |
| Part time | 782 (0.3) | 53 (0.6) | 62 (0.5) | 10 (0.5) | 36 (0.5) | 103 (0.5) | 1,046 (0.3) |  |
| Retired | 14,555 (5.8) | 446 (5.0) | 732 (5.3) | 88 (4.6) | 356 (4.4) | 1,097 (5.1) | 17,274 (5.6) |  |
| Studying/Training | 17 (0) | 5 (0.1) | 10 (0.1) | 2 (0.1) | 5 (0.1) | 4 (0) | 43 (0) |  |
| Unemployed | 820 (0.3) | 102 (1.1) | 117 (0.9) | 16 (1) | 68 (0.8) | 96 (0.5) | 1,219 (0.4) | <0.001 |
| Others | 1,053 (0.4) | 44 (0.5) | 86 (0.6) | 9 (0.5) | 34 (0.4) | 107 (0.5) | 1,333 (0.4) |  |
| Not indicated | 209,959 (83.1) | 7,313 (81.2) | 11,379(82.4) | 1,532(80.8) | 6,723(83.5) | 17,570 (81.5) | 254,476 (82.9) |  |
| Missing | 22,809 (9.0) | 752 (8.4) | 1,165 (8.4) | 193 (10.2) | 660 (8.2) | 2,167 (10.1) | 27,746 (9.0) |  |
|  |  |  |  |  |  |  |  |  |
|  |  |  |  |  |  |  |  |  |
| **Modified Rankin Score (mRS)** |  |  |  |  |  |  |  |  |
| 0 | 129,827 (51.4) | 4,502 (50.0) | 6,205 (44.9) | 1,033(54.5) | 4,171(51.8) | 11,746 (54.5) | 157,484 (51.3) |  |
| 1 | 43,032 (17.0) | 1,497 (16.6) | 2,599 (18.8) | 299 (15.8) | 1,434(17.8) | 3,779 (17.5) | 52,640 (17.1) |  |
| 2 | 28,738 (11.4) | 928 (10.3) | 1,633 (11.8) | 179 (9.5) | 809 (10.1) | 2,121 (9.8) | 34,408 (11.2) | <0.001 |
| 3 | 31,635 (12.5) | 1,300 (14.4) | 2,183 (15.8) | 239 (12.6) | 998 (12.4) | 2,371 (11.0) | 38,726 (12.6) |  |
| 4 | 15,410 (6.1) | 592 (6.6) | 926 (6.7) | 110 (5.8) | 489 (6.1) | 1,192 (5.5) | 18,719 (6.1) |  |
| 5 | 4,107 (1.6) | 184 (2.0) | 260 (1.9) | 35 (1.9) | 149 (1.9) | 342 (1.6) | 5,077 (1.7) |  |
| **Comorbidities. N (%)** |  |  |  |  |  |  |  |  |
| **Heart Failure** |  |  |  |  |  |  |  |  |
| No | 240,159 (95.0) | 8,475 (94.1) | 12,856(93.1) | 1,776(93.7) | 7,630(94.8) | 20,653 (95.8) | 291,549 (95.0) | <0.001 |
| Yes | 12,590 (5.0) | 528 (5.9) | 950 (6.9) | 119 (6.3) | 420 (5.2) | 898 (4.2) | 15,505 (5.1) |  |
|  |  |  |  |  |  |  |  |  |
| **Hypertension** |  |  |  |  |  |  |  |  |
| No | 112,202 (44.4) | 2,440 (27.1) | 4,394 (31.8) | 672 (35.5) | 3,189(39.6) | 9,259 (43.0) | 132,156 (43.0) | <0.001 |
| Yes | 140,547 (56) | 6,563 (72.9) | 9,412 (68.2) | 1,223(65.5) | 4,861(60.4) | 12,292 (57.0) | 174,898 (57.0) |  |
|  |  |  |  |  |  |  |  |  |
| **Atrial Fibrillation** |  |  |  |  |  |  |  |  |
| No | 200,989 (79.5) | 8005 (89) | 12242 (89) | 1641 (87) | 6919 (86) | 18179 (84) | 247,975 (80.8) | <0.001 |
| Yes | 51,760 (20.5) | 998 (11) | 1564 (11) | 254 (13) | 1131 (14) | 3372 (16) | 59,079 (19.2) |  |
|  |  |  |  |  |  |  |  |  |
| **Diabetes Mellitus** |  |  |  |  |  |  |  |  |
| No | 201,322 (79.7) | 5569 (62) | 7528 (55) | 1275 (67) | 5803 (72) | 16829 (78) | 238,326 (77.6) | <0.001 |
| Yes | 51,427 (20) | 3434 (38) | 6278 (45) | 620 (33) | 2247 (28) | 4722 (22) | 68,728 (22.4) |  |
|  |  |  |  |  |  |  |  |  |
| **Stroke/TIA** |  |  |  |  |  |  |  |  |
| No | 187,823 (74.3) | 6480 (72) | 10173 (74) | 1413 (75) | 6202 (77) | 17392 (81) | 229,483 (74.7) | <0.001 |
| Yes | 64,926 (25.6) | 2523 (28) | 3633 (26) | 482 (25) | 1848 (23) | 4159 (19) | 77,571 (25.3) |  |
|  |  |  |  |  |  |  |  |  |
| **Dementia** |  |  |  |  |  |  |  |  |
| No | 50,107 (19.8) | 2276 (25) | 3358 (24) | 477 (25) | 1933 (24) | 4937 (23) | 63,088 (20.6) |  |
| Yes | 3,996 (1.6) | 179 (2) | 241 (2) | 33 (2) | 137 (2) | 353 (2) | 4,939 (1.6) | <0.001 |
| Unknown | 196,747 (77.8) | 6499 (72) | 10114 (73) | 1369 (72) | 5943 (74) | 16138 (75) | 236,810 (77.1) |  |
| Missing | 1,899 (0.8) | 49 (1) | 93 (1) | 16 (1) | 37 (0) | 123 (1) | 2,217 (0.7) |  |
| **Medications. N(%)** |  |  |  |  |  |  |  |  |
| **Antiplatelets** |  |  |  |  |  |  |  |  |
| No | 34,762 (13.8) | 727 (8) | 1040 (8) | 165 (9) | 772 (10) | 2300 (11) | 39,766 (13.0) |  |
| Yes | 10,658 (4.2) | 168 (2) | 347 (3) | 37 (2) | 213 (3) | 631 (3) | 12,054 (3.9) |  |
| Contraindicated | 6,340 (2.5) | 103 (1) | 177 (1) | 52 (3) | 146 (2) | 441 (2) | 7,259 (3.4) | <0.001 |
| Missing | 200,989(79.5) | 8005 (89) | 12242 (89) | 1641 (87) | 6919 (86) | 18179 (84) | 247,975 (80.8) |  |
|  |  |  |  |  |  |  |  |  |
| **Anticoagulant** |  |  |  |  |  |  |  |  |
| No | 117,310 (46.4) | 4971 (55) | 7274 (53) | 971 (51) | 4265 (53) | 10794 (50) | 145,585 |  |
| Yes | 34,639 (13.7) | 790 (9) | 1124 (8) | 205 (11) | 799 (10) | 2182 (10) | 39,739 (12.9) |  |
| Contraindicated | 13,526 (5.3) | 369 (4) | 921 (7) | 68 (4) | 327 (4) | 1276 (6) | 16,487(5.4) | <0.001 |
| Missing | 87,274 (34.5) | 2873 (32) | 4487 (33) | 651 (34) | 2659 (33) | 7299 (34) | 105,243 (34.3) |  |
|  |  |  |  |  |  |  |  |  |
| **Anticoagulant type **** |  |  |  |  |  |  |  |  |
| DOAC | 18010 (7) | 463 (5) | 601 (4) | 117 (6) | 436 (5) | 1166 (5) | 20,793 (6.8) |  |
| Vitamin K | 5255 (2) | 123 (1) | 204 (1) | 32 (2) | 132 (2) | 328 (2) | 6,074 (2.0) | <0.001 |
| Heparin | 297 (0) | 18 (0) | 24 (0) | 6 (0) | 13 (0) | 23 (0) | 381 (0.1) |  |
|  |  |  |  |  |  |  |  |  |
| **Type of Stroke** |  |  |  |  |  |  |  |  |
| Infarction | 221574 (88) | 7606 (84) | 11645 (84) | 1626 (86) | 6857 (85) | 18634 (86) | 267942 (87.3) |  |
| PIH Hemorrhage | 29943 (12) | 1314 (15) | 2050 (15) | 262 (14) | 1160 (14) | 2790 (13) | 37519 (12.2) | <0.001 |
| Missing | 1232 (0) | 83 (1) | 111 (1) | 7 (0) | 33 (0) | 127 (0) | 1593 (0.5) |  |
|  |  |  |  |  |  |  |  |  |
| **Thrombolysis** |  |  |  |  |  |  |  |  |
| Yes | 31575 (12) | 1019 (11) | 1780 (13) | 196 (10) | 1108 (14) | 3095 (14) | 38773 (12.6) |  |
| No | 3250 (1) | 295 (3) | 386 (3) | 52 (3) | 115 (1) | 318 (1) | 4416 (1.5) | <0.001 |
| Not indicated | 217924 (86) | 7689 (85) | 11640 (84) | 1647 (87) | 6827 (85) | 18138 (84) | 263865 (85.9) |  |
|  |  |  |  |  |  |  |  |  |
| **Any Intra-arterial intervention?** | |  |  |  |  |  |  |  |
| No | 123371 (49) | 4107 (46) | 6327 (46) | 880 (46) | 3722 (46) | 9773 (45) | 148180 (48.3) |  |
| Yes | 3034 (1) | 211 (2) | 265 (2) | 41 (2) | 264 (3) | 648 (3) | 4463 (1.5) | <0.001 |
| Not indicated | 53980 (21) | 1621 (18) | 2496 (18) | 314 (17) | 1481 (18) | 4386 (20) | 64278 (20.9) |  |
| Missing | 72364 (29) | 3064 (34) | 4718 (34) | 660 (35) | 2583 (32) | 6744 (31) | 90133 (29.3) |  |
|  |  |  |  |  |  |  |  |  |
| **Anticoagulation reversal therapy** | |  |  |  |  |  |  |  |
| No | 1809 (1) | 25 (0) | 72 (0) | 9 (0) | 35 (0) | 103 (0) | 2053 (0.7) |  |
| Yes | 2324 (1) | 45 (0) | 88 (1) | 14 (1) | 55 (1) | 163 (1) | 2689 (0.9) | <0.001 |
| Not indicated | 108373 (43) | 3170 (35) | 4989 (36) | 742 (39) | 3059 (38) | 8596 (40) | 128929 (42.0) |  |
| Missing | 140243 (55) | 5763 (64) | 8657 (63) | 1130 (60) | 4901 (61) | 12689 (59) | 173383 (56.5) |  |
|  |  |  |  |  |  |  |  |  |
| **Received Palliative Care** |  |  |  |  |  |  |  |  |
| No | 239811 (95) | 8798 (98) | 13386 (97) | 1833 (97) | 7765 (96) | 20652 (96) | 292245 (95.2) | <0.001 |
| Yes | 12938 (5) | 205 (2) | 420 (3) | 62 (3) | 285 (4) | 899 (4) | 14809 (4.8) |  |
|  |  |  |  |  |  |  |  |  |
| N (%) * = Number (percentage).  ** The total number is from the number of only those on anticoagulant while the (% ) is of the total number of all participants | | | | | | | | |
