# Supplementary material for: Ethnic disparities in the longer-term clinical care and outcomes of stroke patients in England
Source: PLoS One. 2026 Aug 5;21(8):e0354353. doi: 10.1371/journal.pone.0354353 (PMC13440867; doi:10.1371/journal.pone.0354353)
Supplement: Supplementary file 2 [file pone.0354353.s002.docx]

# **Supporting Information**

**S1 Table. Baseline demography, comorbidities, use of medication, and stroke episode outcomes of all the stroke survivors stratified by their ethnicities.**

| **Baseline Variable** | **White**  **N (%) * = 252,749 (82.3)** | **Black**  **N (%) = 9,003 (2.9)** | **Asian**  **N (%) = 13,806 (4.5)** | **Mixed**  **N (%) = 1,895 (0.6)** | **Others**  **N (%) = 8,050 (2.6)** | **Unknown**  **N (%) = 21,551 (7.0)** | **Total Number**  **N (%) = 307,054 (100)** | **P value** |
| --- | --- | --- | --- | --- | --- | --- | --- | --- |
| **Demographics. N (%)** |  |  |  |  |  |  |  |  |
| **Age categories** |  |  |  |  |  |  |  |  |
| <40yrs | 3,618 (1.4) | 334 (3.7) | 522 (3.8) | 98 (5.2) | 331 (4.1) | 516 (2.4) | 5,419 (1.8) |  |
| 40-44yrs | 2,917 (1.2) | 339 (3.8) | 471 (3.4) | 74 (3.9) | 246 (3.1) | 463 (2.2) | 4,510 (1.4) |  |
| 45-49yrs | 5,466 (2.2) | 575 (6.4) | 635 (4.6) | 113 (6.0) | 395 (4.9) | 769 (3.6) | 7,953 (2.6) |  |
| 50-54yrs | 9,287 (3.7) | 887 (9.9) | 875 (6.3) | 153 (8.1) | 584 (7.3) | 1,185 (5) | 12,971 (4.2) |  |
| 55-59yrs | 12,887 (5.1) | 1,059 (11.8) | 1,086 (7.9) | 161 (8.5) | 724 (9.0) | 1,493 (6.9) | 17,410 (5.7) |  |
| 60-64yrs | 16,809 (6.7) | 1,037 (11.5) | 1,520 (11.1) | 197 (10.4) | 830 (10.3) | 1,817 (8.4) | 22,210 (7.2) |  |
| 65-69yrs | 22,198 (8.8) | 849 (9.4) | 1,668 (12.1) | 149 (7.9) | 790 (9.8) | 2,137 (9.9) | 27,791 (9.1) | <0.001 |
| 70-74yrs | 30,669 (12.1) | 823 (9.1) | 1,601 (11.7) | 222 (11.7) | 900 (11.2) | 2,618 (12.2) | 36,833 (12.0) |  |
| 75-79yrs | 37,882 (15.0) | 1,027 (11.4) | 1,822 (13.2) | 226 (11.9) | 979 (12.2) | 2,897 (13.4) | 44,833 (14.6) |  |
| 80-84yrs | 42,703 (16.9) | 1,048 (11.6) | 1,790 (13.0) | 228 (12.0) | 981 (12.2) | 3,047 (14.1) | 49,797 (16.2) |  |
| 85-89yrs | 38,584 (15.3) | 648 (7.2) | 1,214 (8.8) | 187 (9.9) | 751 (9.3) | 2,642 (12.3) | 44,026 (14.3) |  |
| 90+above | 29,729 (11.8) | 377 (4.2) | 602 (4.4) | 87 (5.6) | 539 (6.7) | 1,967 (9.1) | 33,301 (10.9) |  |
|  |  |  |  |  |  |  |  |  |
| **Gender** |  |  |  |  |  |  |  |  |
| Male | 129,235 (51.1) | 4,751 (52.8) | 8,079 (58.5) | 996 (52.6) | 4,524(56.2) | 11,670 (54.2) | 159,255 (51.9) | <0.001 |
| Female | 123,514 (48.9) | 4,252 (47.2) | 5,727 (41.5) | 899 (47.4) | 3,526(43.8) | 9,881 (45.9) | 147,799 (48.1) |  |
|  |  |  |  |  |  |  |  |  |
| **Employment Status** |  |  |  |  |  |  |  |  |
| Full time | 2,754 (1.1) | 288 (3.2) | 255 (1.9) | 45 (2.4) | 168 (2.1) | 407 (1.9) | 3,917 (1.3) |  |
| Part time | 782 (0.3) | 53 (0.6) | 62 (0.5) | 10 (0.5) | 36 (0.5) | 103 (0.5) | 1,046 (0.3) |  |
| Retired | 14,555 (5.8) | 446 (5.0) | 732 (5.3) | 88 (4.6) | 356 (4.4) | 1,097 (5.1) | 17,274 (5.6) |  |
| Studying/Training | 17 (0) | 5 (0.1) | 10 (0.1) | 2 (0.1) | 5 (0.1) | 4 (0) | 43 (0) |  |
| Unemployed | 820 (0.3) | 102 (1.1) | 117 (0.9) | 16 (1) | 68 (0.8) | 96 (0.5) | 1,219 (0.4) | <0.001 |
| Others | 1,053 (0.4) | 44 (0.5) | 86 (0.6) | 9 (0.5) | 34 (0.4) | 107 (0.5) | 1,333 (0.4) |  |
| Not indicated | 209,959 (83.1) | 7,313 (81.2) | 11,379(82.4) | 1,532(80.8) | 6,723(83.5) | 17,570 (81.5) | 254,476 (82.9) |  |
| Missing | 22,809 (9.0) | 752 (8.4) | 1,165 (8.4) | 193 (10.2) | 660 (8.2) | 2,167 (10.1) | 27,746 (9.0) |  |
|  |  |  |  |  |  |  |  |  |
|  |  |  |  |  |  |  |  |  |
| **Modified Rankin Score (mRS)** |  |  |  |  |  |  |  |  |
| 0 | 129,827 (51.4) | 4,502 (50.0) | 6,205 (44.9) | 1,033(54.5) | 4,171(51.8) | 11,746 (54.5) | 157,484 (51.3) |  |
| 1 | 43,032 (17.0) | 1,497 (16.6) | 2,599 (18.8) | 299 (15.8) | 1,434(17.8) | 3,779 (17.5) | 52,640 (17.1) |  |
| 2 | 28,738 (11.4) | 928 (10.3) | 1,633 (11.8) | 179 (9.5) | 809 (10.1) | 2,121 (9.8) | 34,408 (11.2) | <0.001 |
| 3 | 31,635 (12.5) | 1,300 (14.4) | 2,183 (15.8) | 239 (12.6) | 998 (12.4) | 2,371 (11.0) | 38,726 (12.6) |  |
| 4 | 15,410 (6.1) | 592 (6.6) | 926 (6.7) | 110 (5.8) | 489 (6.1) | 1,192 (5.5) | 18,719 (6.1) |  |
| 5 | 4,107 (1.6) | 184 (2.0) | 260 (1.9) | 35 (1.9) | 149 (1.9) | 342 (1.6) | 5,077 (1.7) |  |
| **Comorbidities. N (%)** |  |  |  |  |  |  |  |  |
| **Heart Failure** |  |  |  |  |  |  |  |  |
| No | 240,159 (95.0) | 8,475 (94.1) | 12,856(93.1) | 1,776(93.7) | 7,630(94.8) | 20,653 (95.8) | 291,549 (95.0) | <0.001 |
| Yes | 12,590 (5.0) | 528 (5.9) | 950 (6.9) | 119 (6.3) | 420 (5.2) | 898 (4.2) | 15,505 (5.1) |  |
|  |  |  |  |  |  |  |  |  |
| **Hypertension** |  |  |  |  |  |  |  |  |
| No | 112,202 (44.4) | 2,440 (27.1) | 4,394 (31.8) | 672 (35.5) | 3,189(39.6) | 9,259 (43.0) | 132,156 (43.0) | <0.001 |
| Yes | 140,547 (56) | 6,563 (72.9) | 9,412 (68.2) | 1,223(65.5) | 4,861(60.4) | 12,292 (57.0) | 174,898 (57.0) |  |
|  |  |  |  |  |  |  |  |  |
| **Atrial Fibrillation** |  |  |  |  |  |  |  |  |
| No | 200,989 (79.5) | 8005 (89) | 12242 (89) | 1641 (87) | 6919 (86) | 18179 (84) | 247,975 (80.8) | <0.001 |
| Yes | 51,760 (20.5) | 998 (11) | 1564 (11) | 254 (13) | 1131 (14) | 3372 (16) | 59,079 (19.2) |  |
|  |  |  |  |  |  |  |  |  |
| **Diabetes Mellitus** |  |  |  |  |  |  |  |  |
| No | 201,322 (79.7) | 5569 (62) | 7528 (55) | 1275 (67) | 5803 (72) | 16829 (78) | 238,326 (77.6) | <0.001 |
| Yes | 51,427 (20) | 3434 (38) | 6278 (45) | 620 (33) | 2247 (28) | 4722 (22) | 68,728 (22.4) |  |
|  |  |  |  |  |  |  |  |  |
| **Stroke/TIA** |  |  |  |  |  |  |  |  |
| No | 187,823 (74.3) | 6480 (72) | 10173 (74) | 1413 (75) | 6202 (77) | 17392 (81) | 229,483 (74.7) | <0.001 |
| Yes | 64,926 (25.6) | 2523 (28) | 3633 (26) | 482 (25) | 1848 (23) | 4159 (19) | 77,571 (25.3) |  |
|  |  |  |  |  |  |  |  |  |
| **Dementia** |  |  |  |  |  |  |  |  |
| No | 50,107 (19.8) | 2276 (25) | 3358 (24) | 477 (25) | 1933 (24) | 4937 (23) | 63,088 (20.6) |  |
| Yes | 3,996 (1.6) | 179 (2) | 241 (2) | 33 (2) | 137 (2) | 353 (2) | 4,939 (1.6) | <0.001 |
| Unknown | 196,747 (77.8) | 6499 (72) | 10114 (73) | 1369 (72) | 5943 (74) | 16138 (75) | 236,810 (77.1) |  |
| Missing | 1,899 (0.8) | 49 (1) | 93 (1) | 16 (1) | 37 (0) | 123 (1) | 2,217 (0.7) |  |
| **Medications. N(%)** |  |  |  |  |  |  |  |  |
| **Antiplatelets** |  |  |  |  |  |  |  |  |
| No | 34,762 (13.8) | 727 (8) | 1040 (8) | 165 (9) | 772 (10) | 2300 (11) | 39,766 (13.0) |  |
| Yes | 10,658 (4.2) | 168 (2) | 347 (3) | 37 (2) | 213 (3) | 631 (3) | 12,054 (3.9) |  |
| Contraindicated | 6,340 (2.5) | 103 (1) | 177 (1) | 52 (3) | 146 (2) | 441 (2) | 7,259 (3.4) | <0.001 |
| Missing | 200,989(79.5) | 8005 (89) | 12242 (89) | 1641 (87) | 6919 (86) | 18179 (84) | 247,975 (80.8) |  |
|  |  |  |  |  |  |  |  |  |
| **Anticoagulant** |  |  |  |  |  |  |  |  |
| No | 117,310 (46.4) | 4971 (55) | 7274 (53) | 971 (51) | 4265 (53) | 10794 (50) | 145,585 |  |
| Yes | 34,639 (13.7) | 790 (9) | 1124 (8) | 205 (11) | 799 (10) | 2182 (10) | 39,739 (12.9) |  |
| Contraindicated | 13,526 (5.3) | 369 (4) | 921 (7) | 68 (4) | 327 (4) | 1276 (6) | 16,487(5.4) | <0.001 |
| Missing | 87,274 (34.5) | 2873 (32) | 4487 (33) | 651 (34) | 2659 (33) | 7299 (34) | 105,243 (34.3) |  |
|  |  |  |  |  |  |  |  |  |
| **Anticoagulant type **** |  |  |  |  |  |  |  |  |
| DOAC | 18010 (7) | 463 (5) | 601 (4) | 117 (6) | 436 (5) | 1166 (5) | 20,793 (6.8) |  |
| Vitamin K | 5255 (2) | 123 (1) | 204 (1) | 32 (2) | 132 (2) | 328 (2) | 6,074 (2.0) | <0.001 |
| Heparin | 297 (0) | 18 (0) | 24 (0) | 6 (0) | 13 (0) | 23 (0) | 381 (0.1) |  |
|  |  |  |  |  |  |  |  |  |
| **Type of Stroke** |  |  |  |  |  |  |  |  |
| Infarction | 221574 (88) | 7606 (84) | 11645 (84) | 1626 (86) | 6857 (85) | 18634 (86) | 267942 (87.3) |  |
| PIH Hemorrhage | 29943 (12) | 1314 (15) | 2050 (15) | 262 (14) | 1160 (14) | 2790 (13) | 37519 (12.2) | <0.001 |
| Missing | 1232 (0) | 83 (1) | 111 (1) | 7 (0) | 33 (0) | 127 (0) | 1593 (0.5) |  |
|  |  |  |  |  |  |  |  |  |
| **Thrombolysis** |  |  |  |  |  |  |  |  |
| Yes | 31575 (12) | 1019 (11) | 1780 (13) | 196 (10) | 1108 (14) | 3095 (14) | 38773 (12.6) |  |
| No | 3250 (1) | 295 (3) | 386 (3) | 52 (3) | 115 (1) | 318 (1) | 4416 (1.5) | <0.001 |
| Not indicated | 217924 (86) | 7689 (85) | 11640 (84) | 1647 (87) | 6827 (85) | 18138 (84) | 263865 (85.9) |  |
|  |  |  |  |  |  |  |  |  |
| **Any Intra-arterial intervention?** | |  |  |  |  |  |  |  |
| No | 123371 (49) | 4107 (46) | 6327 (46) | 880 (46) | 3722 (46) | 9773 (45) | 148180 (48.3) |  |
| Yes | 3034 (1) | 211 (2) | 265 (2) | 41 (2) | 264 (3) | 648 (3) | 4463 (1.5) | <0.001 |
| Not indicated | 53980 (21) | 1621 (18) | 2496 (18) | 314 (17) | 1481 (18) | 4386 (20) | 64278 (20.9) |  |
| Missing | 72364 (29) | 3064 (34) | 4718 (34) | 660 (35) | 2583 (32) | 6744 (31) | 90133 (29.3) |  |
|  |  |  |  |  |  |  |  |  |
| **Anticoagulation reversal therapy** | |  |  |  |  |  |  |  |
| No | 1809 (1) | 25 (0) | 72 (0) | 9 (0) | 35 (0) | 103 (0) | 2053 (0.7) |  |
| Yes | 2324 (1) | 45 (0) | 88 (1) | 14 (1) | 55 (1) | 163 (1) | 2689 (0.9) | <0.001 |
| Not indicated | 108373 (43) | 3170 (35) | 4989 (36) | 742 (39) | 3059 (38) | 8596 (40) | 128929 (42.0) |  |
| Missing | 140243 (55) | 5763 (64) | 8657 (63) | 1130 (60) | 4901 (61) | 12689 (59) | 173383 (56.5) |  |
|  |  |  |  |  |  |  |  |  |
| **Received Palliative Care** |  |  |  |  |  |  |  |  |
| No | 239811 (95) | 8798 (98) | 13386 (97) | 1833 (97) | 7765 (96) | 20652 (96) | 292245 (95.2) | <0.001 |
| Yes | 12938 (5) | 205 (2) | 420 (3) | 62 (3) | 285 (4) | 899 (4) | 14809 (4.8) |  |
|  |  |  |  |  |  |  |  |  |
| N (%) * = Number (percentage).  ** The total number is from the number of only those on anticoagulant while the (% ) is of the total number of all participants | | | | | | | | |

**S2 Table. Baseline Characteristics and Clinical Outcomes in only stroke survivors who attended their 6 months follow up according to their ethnicities.**

| **Baseline Variable** | **White**  **N (%)* = 66,967 (26.5)** | **Black**  **N (%) = 2,457 (27.3)** | **Asian**  **N (%) = 3,598 (26.1)** | **Mixed**  **N (%) = 522 (27.6)** | **Others**  **N (%) = 1,888 (23.4)** | **Unknown**  **N (%) = 4,981 (23.1)** | **Total Number**  **N (%) = 80,413 (26.2)** | **P value** |
| --- | --- | --- | --- | --- | --- | --- | --- | --- |
| **Demographics. N (%)** |  |  |  |  |  |  |  |  |
| **Age categories** |  |  |  |  |  |  |  |  |
| <40yrs | 899 (1) | 80 (3) | 117 (3) | 23 (4) | 64 (3) | 119 (2) | 1,302 (1.6) |  |
| 40-44yrs | 806 (1) | 78 (3) | 127 (4) | 21 (4) | 60 (3) | 108 (2) | 1,200 (1.5) |  |
| 45-49yrs | 1602 (2) | 168 (7) | 158 (4) | 31 (6) | 94 (5) | 183 (4) | 2,236 (2.8) |  |
| 50-54yrs | 2750 (4) | 243 (10) | 235 (7) | 45 (9) | 144 (8) | 302 (6) | 3,719 (4.6) |  |
| 55-59yrs | 4031 (6) | 328 (13) | 298 (8) | 44 (8) | 197 (10) | 404 (8) | 5,302 (6.9) |  |
| 60-64yrs | 5390 (8) | 317 (13) | 421 (12) | 68 (13) | 227 (12) | 441 (9) | 6,864 (8.5) |  |
| 65-69yrs | 6987 (10) | 249 (10) | 492 (14) | 38 (7) | 198 (10) | 550 (11) | 8,514 (10.6) | <0.001 |
| 70-74yrs | 9737 (15) | 210 (9) | 428 (12) | 59 (11) | 244 (13) | 710 (14) | 11,388 (14.2) |  |
| 75-79yrs | 11169 (17) | 256 (10) | 471 (13) | 68 (13) | 202 (11) | 733 (15) | 12,899 (16.0) |  |
| 80-84yrs | 11159 (17) | 277 (11) | 460 (13) | 59 (11) | 224 (12) | 676 (14) | 12,855 (16.0) |  |
| 85-89yrs | 8124 (12) | 169 (7) | 284 (8) | 52 (10) | 154 (8) | 490 (10) | 9,273 (11.5) |  |
| 90+above | 4313 (6) | 82 (3) | 107 (3) | 14 (3) | 80 (4) | 265 (5) | 4,861 (6.1) |  |
|  |  |  |  |  |  |  |  |  |
| **Gender** |  |  |  |  |  |  |  |  |
| Male | 36104 (54) | 1278 (52) | 2097 (58) | 263 (50) | 1110 (59) | 2813 (56) | 43,665 (54.3) | <0.001 |
| Female | 30863 (46) | 1179 (48) | 1501 (42) | 259 (50) | 778 (41) | 2168 (44) | 36,748 (45.7) |  |
|  |  |  |  |  |  |  |  |  |
| **Employment Status** |  |  |  |  |  |  |  |  |
| Full time | 2754 (4) | 288 (12) | 255 (7) | 45 (9) | 168 (9) | 407 (8) | 3,917 (4.9) |  |
| Part time | 782 (1) | 53 (2) | 62 (2) | 10 (2) | 36 (2) | 103 (2) | 1,046 (1.3) |  |
| Retired | 14555 (22) | 446 (18) | 732 (20) | 88 (17) | 356 (19) | 1097 (22) | 17,274 (21.5) |  |
| Studying/Training | 17 (0) | 5 (0) | 10 (0) | 2 (0) | 5 (0) | 4 (0) | 43 (0.1) |  |
| Unemployed | 820 (1) | 102 (4) | 117 (3) | 16 (3) | 68 (4) | 96 (2) | 1,219 (1.5) | <0.001 |
| Others | 1053 (2) | 44 (2) | 86 (2) | 9 (2) | 34 (2) | 107 (2) | 1,333 (1.7) |  |
| Not indicated | 46367 (69) | 1488 (61) | 2294 (64) | 344 (66) | 1189 (63) | 3071 (62) | 54,753 (68.1) |  |
| Missing | 619 (1) | 31 (1) | 42 (1) | 8 (2) | 32 (2) | 96 (2) | 828 (1.0) |  |
|  |  |  |  |  |  |  |  |  |
|  |  |  |  |  |  |  |  |  |
| **Rankin Score, N (%)** |  |  |  |  |  |  |  |  |
| 0 | 39092 (58) | 1305 (53) | 1608 (45) | 300 (57) | 1011 (54) | 2997 (60) | 46,313 (57.6) |  |
| 1 | 12575 (19) | 408 (17) | 766 (21) | 92 (18) | 379 (20) | 977 (20) | 15,197 (18.9) |  |
| 2 | 6921 (10) | 274 (11) | 475 (13) | 47 (9) | 188 (10) | 468 (9) | 8,373 (10.4) | <0.001 |
| 3 | 6023 (9) | 312 (13) | 543 (15) | 64 (12) | 213 (11) | 393 (8) | 8,373 (9.4) |  |
| 4 | 1984 (3) | 128 (5) | 163 (5) | 13 (2) | 76 (4) | 115 (2) | 2,479 (3.1) |  |
| 5 | 372 (1) | 30 (1) | 43 (1) | 6 (1) | 21 (1) | 31 (1) | 503 (0.6) |  |
| **Comorbidities. N (%)** |  |  |  |  |  |  |  |  |
| **Heart Failure** |  |  |  |  |  |  |  |  |
| No | 64456 (96) | 2334 (95) | 3406 (95) | 500 (96) | 1799 (95) | 4836 (97) | 77,331 (96.2) | <0.001 |
| Yes | 2511 (4) | 123 (5) | 192 (5) | 22 (4) | 89 (5) | 145 (3) | 3,082 (3.8) |  |
|  |  |  |  |  |  |  |  |  |
| **Hypertension** |  |  |  |  |  |  |  |  |
| No | 28985 (43) | 610 (25) | 1177 (33) | 169 (32) | 700 (37) | 2035 (41) | 33,676 (41.9) | <0.001 |
| Yes | 37982 (57) | 1847 (75) | 2421 (67) | 353 (68) | 1188 (63) | 2946 (59) | 46,737 (58.1) |  |
|  |  |  |  |  |  |  |  |  |
| **Atrial Fibrillation** |  |  |  |  |  |  |  |  |
| No | 55994 (84) | 2227 (91) | 3248 (90) | 472 (90) | 1688 (89) | 4373 (88) | 68,002 (84.6) | <0.001 |
| Yes | 10973 (16) | 230 (9) | 350 (10) | 50 (10) | 200 (11) | 608 (12) | 12,411 (15.4) |  |
|  |  |  |  |  |  |  |  |  |
| **Diabetes Mellitus** |  |  |  |  |  |  |  |  |
| No | 53228 (79) | 1478 (60) | 1974 (55) | 347 (66) | 1331 (70) | 3972 (80) | 62,330 (77.5) | <0.001 |
| Yes | 13739 (21) | 979 (40) | 1624 (45) | 175 (34) | 557 (30) | 1009 (20) | 18,083 (22.5) |  |
|  |  |  |  |  |  |  |  |  |
| **Stroke/TIA** |  |  |  |  |  |  |  |  |
| No | 51014 (76) | 1813 (74) | 2753 (77) | 390 (75) | 1488 (79) | 4144 (83) | 61,602 (76.6) | <0.001 |
| Yes | 15953 (24) | 644 (26) | 845 (23) | 132 (25) | 400 (21) | 837 (17) | 18,811 (23.4) |  |
|  |  |  |  |  |  |  |  |  |
| **Dementia** |  |  |  |  |  |  |  |  |
| No | 14875 (22) | 673 (27) | 902 (25) | 125 (24) | 489 (26) | 1376 (28) | 18,440 (22.9) |  |
| Yes | 616 (1) | 44 (2) | 54 (2) | 7 (1) | 19 (1) | 52 (1) | 792 (1.0) | <0.001 |
| Unknown | 51078 (76) | 1732 (70) | 2630 (73) | 387 (74) | 1369 (73) | 3522 (71) | 60,718 (75.5) |  |
| Missing | 398 (1) | 8 (0) | 12 (0) | 3 (1) | 11 (1) | 31 (1) | 463 (0.6) |  |
| **Medications. N (%)** |  |  |  |  |  |  |  |  |
| **Antiplatelets** |  |  |  |  |  |  |  |  |
| No | 7574 (11) | 170 (7) | 238 (7) | 34 (7) | 151 (8) | 427 (9) | 8,594 (10.7) |  |
| Yes | 2117 (3) | 34 (1) | 80 (2) | 5 (1) | 29 (2) | 108 (2) | 2,373 (3.0) |  |
| Contraindicated | 1282 (2) | 26 (1) | 32 (1) | 11 (2) | 20 (1) | 73 (1) | 1,444 (1.8) | <0.001 |
| Missing | 55994 (84) | 2227 (91) | 3248 (90) | 472 (90) | 1688 (89) | 4373 (88) | 68,002 (84.6) |  |
|  |  |  |  |  |  |  |  |  |
| **Anticoagulant** |  |  |  |  |  |  |  |  |
| No | 34802 (52) | 1465 (60) | 2068 (57) | 288 (55) | 1121 (59) | 2946 (59) | 42,690 (53.1) |  |
| Yes | 7995 (12) | 193 (8) | 250 (7) | 51 (10) | 150 (8) | 395 (8) | 9,034 (11.2) |  |
| Contraindicated | 3737 (6) | 162 (7) | 328 (9) | 22 (4) | 87 (5) | 350 (7) | 4,686 (5.8) | <0.001 |
| Missing | 20433 (31) | 637 (26) | 952 (26) | 161 (31) | 530 (28) | 1290 (26) | 24,003 (29.9) |  |
|  |  |  |  |  |  |  |  |  |
| **Anticoagulant type**** |  |  |  |  |  |  |  |  |
| DOAC | 4351 (6) | 113 (5) | 133 (4) | 25 (5) | 93 (5) | 242 (5) | 4,957 (6.2) |  |
| Vitamin K | 1412 (2) | 36 (1) | 54 (2) | 14 (3) | 29 (2) | 64 (1) | 1,609 (2.0) | <0.001 |
| Heparin | 68 (0) | 2 (0) | 6 (0) | 1 (0) | 2 (0) | 2 (0) | 81 (0.1) |  |
|  |  |  |  |  |  |  |  |  |
| **Stroke Episode Outcomes. N (%)** | |  |  |  |  |  |  |  |
| **Day of Occurrence** |  |  |  |  |  |  |  |  |
| Sunday | 9206 (14) | 342 (14) | 503 (14) | 74 (14) | 261 (14) | 648 (13) | 11034 (13.7) |  |
| Monday | 9833 (15) | 353 (14) | 503 (14) | 68 (13) | 305 (16) | 712 (14) | 11774 (14.6) |  |
| Tuesday | 9710 (14) | 359 (15) | 506 (14) | 76 (15) | 276 (15) | 737 (15) | 11664 (14.5) |  |
| Wednesday | 9657 (14) | 358 (15) | 559 (16) | 73 (14) | 269 (14) | 744 (15) | 11660 (14.5) | 0.749 |
| Thursday | 9593 (14) | 364 (15) | 478 (13) | 76 (15) | 277 (15) | 718 (14) | 11506 (14.3) |  |
| Friday | 9734 (15) | 339 (14) | 532 (15) | 88 (17) | 245 (13) | 750 (15) | 11688 (14.5) |  |
| Saturday | 9234 (14) | 342 (14) | 517 (14) | 67 (13) | 255 (14) | 672 (13) | 11087 (13.9) |  |
|  |  |  |  |  |  |  |  |  |
| **Time of Occurrence** |  |  |  |  |  |  |  |  |
| 0000-0400hrs | 3461 (5) | 174 (7) | 296 (8) | 31 (6) | 172 (9) | 304 (6) | 4438 (5.5) |  |
| 0400-0800hrs | 7312 (11) | 263 (11) | 364 (10) | 69 (13) | 202 (11) | 575 (12) | 8785 (10.9) |  |
| 0800-1200hrs | 14046 (21) | 461 (19) | 719 (20) | 97 (19) | 400 (21) | 973 (20) | 16696 (20.8) |  |
| 1200-1600hrs | 9758 (15) | 310 (13) | 533 (15) | 78 (15) | 280 (15) | 734 (15) | 11693 (14.5) | <0.001 |
| 1600-2000hrs | 8172 (12) | 331 (13) | 457 (13) | 64 (12) | 249 (13) | 613 (12) | 9886 (12.3) |  |
| 2000-2400hrs | 6270 (9) | 237 (10) | 304 (8) | 36 (7) | 182 (10) | 428 (9) | 7457 (9.3) |  |
| Unknown | 17948 (27) | 681 (28) | 925 (26) | 147 (28) | 403 (21) | 1354 (27) | 21458 (26.7) |  |
|  |  |  |  |  |  |  |  |  |
| **Precision of Onset Date** |  |  |  |  |  |  |  |  |
| Best estimate | 13189 (20) | 628 (26) | 780 (22) | 148 (28) | 551 (29) | 980 (20) | 16276 (20.2) |  |
| While sleeping | 9155 (14) | 257 (10) | 352 (10) | 40 (8) | 223 (12) | 553 (11) | 10580 (13.2) | <0.001 |
| Precise date | 44623 (67) | 1572 (64) | 2466 (69) | 334 (64) | 1114 (59) | 3448 (69) | 53557 (66.6) |  |
|  |  |  |  |  |  |  |  |  |
| **Precision of Onset Time,** |  |  |  |  |  |  |  |  |
| Best estimate | 24412 (36) | 1012 (41) | 1312 (36) | 229 (44) | 897 (48) | 1778 (36) | 29640 (36.9) |  |
| Unknown | 17948 (27) | 681 (28) | 925 (26) | 147 (28) | 403 (21) | 1354 (27) | 21458 (26.7) | <0.001 |
| Precise | 24607 (37) | 764 (31) | 1361 (38) | 146 (28) | 588 (31) | 1849 (37) | 29315 (36.5) |  |
|  |  |  |  |  |  |  |  |  |
| **Place of Onset** |  |  |  |  |  |  |  |  |
| Hospital | 64123 (96) | 2357 (96) | 3434 (95) | 501 (96) | 1837 (97) | 4826 (97) | 77078 (95.9) | <0.001 |
| Other | 2844 (4) | 100 (4) | 164 (5) | 21 (4) | 51 (3) | 155 (3) | 3335 (4.2) |  |
|  |  |  |  |  |  |  |  |  |
| **Time of Arrival to Hospital** |  |  |  |  |  |  |  |  |
| 0000-0400hrs | 3468 (5) | 185 (8) | 264 (7) | 39 (7) | 166 (9) | 340 (7) | 4462 (5.6) |  |
| 0400-0800hrs | 3666 (5) | 154 (6) | 214 (6) | 30 (6) | 130 (7) | 291 (6) | 4485 (5.6) |  |
| 0800-1200hrs | 18113 (27) | 508 (21) | 782 (22) | 101 (19) | 367 (19) | 1183 (24) | 21054 (26.2) | <0.001 |
| 1200-1600hrs | 18886 (28) | 589 (24) | 936 (26) | 140 (27) | 457 (24) | 1324 (27) | 22332 (27.8) |  |
| 1600-2000hrs | 14167 (21) | 592 (24) | 805 (22) | 121 (23) | 421 (22) | 1102 (22) | 17208 (21.4) |  |
| 2000-2400hrs | 8667 (13) | 429 (17) | 597 (17) | 91 (17) | 347 (18) | 741 (15) | 10872 (13.5) |  |
|  |  |  |  |  |  |  |  |  |
| **Ward of Arrival** |  |  |  |  |  |  |  |  |
| Stroke unit | 55653 (83) | 1921 (78) | 2947 (82) | 415 (80) | 1596 (85) | 4182 (84) | 66714 (83.0) |  |
| MAU/AAU/CDU | 7611 (11) | 296 (12) | 378 (11) | 66 (13) | 170 (9) | 414 (8) | 8935 (11.1) | <0.001 |
| ITU/CCU/HDU | 1106 (2) | 104 (4) | 88 (2) | 20 (4) | 49 (3) | 149 (3) | 1516 (1.9) |  |
| Others | 2597 (4) | 136 (6) | 185 (5) | 21 (4) | 73 (4) | 236 (5) | 3248 (4.0) |  |
|  |  |  |  |  |  |  |  |  |
| **Type of Stroke** |  |  |  |  |  |  |  |  |
| Infarction | 60666 (91) | 2091 (85) | 3101 (86) | 454 (87) | 1625 (86) | 4389 (88) | 72326 (89.9) |  |
| PIH Hemorrhage | 6089 (9) | 348 (14) | 468 (13) | 65 (12) | 259 (14) | 575 (12) | 7804 (9.7) | <0.001 |
| Missing | 212 (0) | 18 (1) | 29 (1) | 3 (1) | 4 (0) | 17 (0) | 283 (0.4) |  |
|  |  |  |  |  |  |  |  |  |
| **Thrombolysis** |  |  |  |  |  |  |  |  |
| Yes | 9048 (14) | 277 (11) | 505 (14) | 41 (8) | 276 (15) | 759 (15) | 10906 (13.6) |  |
| No | 627 (1) | 94 (4) | 107 (3) | 13 (2) | 24 (1) | 61 (1) | 926 (1.2) | <0.001 |
| Not indicated | 57292 (86) | 2086 (85) | 2986 (83) | 468 (90) | 1588 (84) | 4161 (84) | 68581 (85.3) |  |
|  |  |  |  |  |  |  |  |  |
| **Haemorrhage post thrombolysis** | |  |  |  |  |  |  |  |
| No | 5448 (8) | 202 (8) | 340 (9) | 26 (5) | 187 (10) | 507 (10) | 6710 (8.3) |  |
| Yes | 427 (1) | 19 (1) | 18 (1) | 8 (2) | 11 (1) | 49 (1) | 532 (0.7) | <0.001 |
| Not indicated | 24182 (36) | 698 (28) | 1056 (29) | 176 (34) | 572 (30) | 1467 (29) | 28151 (35) |  |
| Missing | 36910 (55) | 1538 (63) | 2184 (61) | 312 (60) | 1118 (59) | 2958 (59) | 45020 (56.0) |  |
|  |  |  |  |  |  |  |  |  |
| **Any Intra-arterial intervention** | |  |  |  |  |  |  |  |
| No | 35779 (53) | 1197 (49) | 1787 (50) | 262 (50) | 966 (51) | 2441 (49) | 42432 (52.8) |  |
| Yes | 911 (1) | 68 (3) | 68 (2) | 13 (2) | 62 (3) | 167 (3) | 1289 (1.6) | <0.001 |
| Not indicated | 11213 (17) | 299 (12) | 508 (14) | 64 (12) | 233 (12) | 674 (14) | 12991 (16.2) |  |
| Missing | 19064 (28) | 893 (36) | 1235 (34) | 183 (35) | 627 (33) | 1699 (34) | 23701 (29.5) |  |
|  |  |  |  |  |  |  |  |  |
| **Anticoagulation reversal therapy** | |  |  |  |  |  |  |  |
| No | 295 (0) | 5 (0) | 15 (0) | 2 (0) | 5 (0) | 11 (0) | 333 (0.4) |  |
| Yes | 442 (1) | 11 (0) | 13 (0) | 2 (0) | 9 (0) | 24 (0) | 501 (0.6) | <0.001 |
| Not indicated | 24182 (36) | 698 (28) | 1056 (29) | 176 (34) | 572 (30) | 1467 (29) | 28151 (35.0) |  |
| Missing | 42048 (63) | 1743 (71) | 2514 (70) | 342 (66) | 1302 (69) | 3479 (70) | 51428 (64.0) |  |
|  |  |  |  |  |  |  |  |  |
| **Received Palliative Care** | |  |  |  |  |  |  |  |
| No | 66834 (100) | 2453 (100) | 3593 (100) | 522 (100) | 1885 (100) | 4978 (100) | 80265 (99.8) | 0.268 |
| Yes | 133 (0) | 4 (0) | 5 (0) | 0 (0) | 3 (0) | 3 (0) | 148 (0.2) |  |
|  |  |  |  |  |  |  |  |  |
| **Comorbidities while on admission** | |  |  |  |  |  |  |  |
| **New Atrial Fibrillation** | |  |  |  |  |  |  |  |
| No | 33238 (50) | 1534 (62) | 2235 (62) | 302 (58) | 1105 (59) | 2890 (58) | 41304 (51.4) |  |
| Yes | 2321 (3) | 56 (2) | 61 (2) | 9 (2) | 53 (3) | 193 (4) | 2693 (3.4) | <0.001 |
| Not indicated | 24182 (36) | 698 (28) | 1056 (29) | 176 (34) | 572 (30) | 1467 (29) | 28151 (35.0) |  |
| Missing | 7226 (11) | 169 (7) | 246 (7) | 35 (7) | 158 (8) | 431 (9) | 8265 (10.3) |  |
|  |  |  |  |  |  |  |  |  |
| **Pneumonia, N (%)** |  |  |  |  |  |  |  |  |
| No | 63582 (95) | 2321 (94) | 3442 (96) | 486 (93) | 1779 (94) | 4727 (95) | 76337 (94.9) |  |
| Yes | 3170 (5) | 126 (5) | 142 (4) | 35 (7) | 107 (6) | 237 (5) | 3817 (4.8) | 0.057 |
| Unknown | 215 (0) | 10 (0) | 14 (0) | 1 (0) | 2 (0) | 17 (0) | 259 (0.3) |  |
|  |  |  |  |  |  |  |  |  |
| **Urinary Tract Infection** |  |  |  |  |  |  |  |  |
| No | 63882 (95) | 2380 (97) | 3495 (97) | 504 (97) | 1839 (97) | 4800 (96) | 76900 (95.6) |  |
| Yes | 2867 (4) | 68 (3) | 91 (3) | 16 (3) | 46 (2) | 165 (3) | 3253 (4.1) | <0.001 |
| Unknown | 218 (0) | 9 (0) | 12 (0) | 2 (0) | 3 (0) | 16 (0) | 260 (0.3) |  |
|  |  |  |  |  |  |  |  |  |
| **COVID-19 Infection, N (%)** |  |  |  |  |  |  |  |  |
| No | 13147 (20) | 627 (26) | 863 (24) | 118 (23) | 464 (25) | 1216 (24) | 16435 (20.4) |  |
| Yes | 661 (1) | 40 (2) | 27 (1) | 5 (1) | 21 (1) | 63 (1) | 817 (1.0) |  |
| Not known | 1683 (3) | 50 (2) | 66 (2) | 9 (2) | 23 (1) | 149 (3) | 1980 (2.5) | <0.001 |
| Not applicable | 50638 (76) | 1696 (69) | 2601 (72) | 383 (73) | 1349 (71) | 3469 (70) | 60136 (74.8) |  |
| Missing | 838 (1) | 44 (2) | 41 (1) | 7 (1) | 31 (2) | 84 (2) | 1045 (1.3) |  |
|  |  |  |  |  |  |  |  |  |
| **Continuous Variables. Mean (SD)** | |  |  |  |  |  |  |  |
| **Time to imaging (min)** | 204.4 (1070.6) | 256.1 (1878.8) | 212.2 (1250.4) | 232.7 (823.5) | 154.9 (514.1) | 157.3 (714.1) | 202.3 (1083.9) | 0.002 |
| **Systolic BP on arrival (mmHg)** | 167.9 (30.6) | 174.0 (33.3) | 172.0 (32.3) | 180.4 (36.1) | 172.8 (30.2) | 172.1 (33.1) | 169.1 (31.1) | <0.001 |
| **Arrival to lowering of BP (Mins)** | 185.7 (323.7) | 17.8 (1038.4) | 116.4 (181.8) | 118.1 (259.4) | 132.7 (293.8) | 117.0 (206.7) | 160.9 (397.1) | 0.013 |
| **Arrival to achieving low SBP (min)** | 446.5 (343.2) | 415.0 (292.5) | 369.8 (314.0) | 447.4 (316.1) | 335.2 (303.2) | 371.8 (304.9) | 429.5 (335.6) | 0.009 |
| **Arrival to Nurse’s assessment (min)** | 222.4 (513.7) | 275.6 (602.4) | 236.8 (558.5) | 290.2 (617.6) | 198.7 (469.4) | 173.2 (422.8) | 221.5 (513.5) | 0.001 |
| **Arrival to Consultant’s assessment (min)** | 600.3 (741.5) | 608.3 (733.6) | 587.9 (710.2) | 650.9 (746.3) | 556.0 (674.3) | 555.1 (671.7) | 596.4 (734.3) | <0.001 |
| **Arrival to mood screening (Days)** | 2.3 (6.2) | 2.3 (7.3) | 1.8 (5.5) | 2.6 (7.7) | 1.8 (6.3) | 1.5 (4.6) | 2.2 (6.1) | <0.001 |
| **Arrival to cognition screening (Days)** | 2.3 (6.4) | 2.2 (6.7) | 1.6 (4.9) | 2.4 (5.8) | 2.0 (7.0) | 1.5 (5.0) | 2.2 (6.3) | <0.001 |
| **NIHSS** score on Arrival** | 5.3 (5.5) | 6.7 (6.3) | 6.1 (5.8) | 5.8 (5.9) | 6.2 (5.9) | 5.9 (5.8) | 5.4 (5.6) | 0.003 |
| **NIHSS score after 24hrs** | 5.6 (6.1) | 7.7 (6.8) | 6.4 (6.4) | 7.5 (6.5) | 7.3 (7.4) | 6.4 (6.5) | 5.8 (6.2) | 0.005 |
| **Arrival Time to thrombolysis (mins)** | 8.1 (25.0) | 6.3 (22.0) | 6.7 (22.4) | 4.6 (19.2) | 6.8 (20.6) | 7.7 (22.9) | 7.9 (24.6) | <0.001 |
| **Time to discharge from stroke unit (min)** | 23942.4 (35036.1) | 33519.1 (41579.4) | 23761.4 (31837.9) | 27910.4 (34086.4) | 28062.6 (38837.2) | 26181.7 (35509.0) | 24479.7 (35274.8) | 0.002 |
| **Time to discharge from Hospital (min)** | 24493.8 (35994.2) | 34744.5 (42838.0) | 24607.4 (32769.8) | 28984.1 (35546.5) | 29965.4 (40727.8) | 27498.5 (37914.0) | 25155 (36377.6) | 0.003 |
|  |  |  |  |  |  |  |  |  |
| ** Computed by the number of patients in each ethnic group that attended the follow up divided by the total number that had stroke x 100%*  **** *The National Institutes of Health Stroke Scale (NHISS) score on arrival was used as a marker of severity of the stroke episode* | | | | | | | | |
