# Supplementary material for: Ethnic disparities in the longer-term clinical care and outcomes of stroke patients in England
Source: PLoS One. 2026 Aug 5;21(8):e0354353. doi: 10.1371/journal.pone.0354353 (PMC13440867; doi:10.1371/journal.pone.0354353)
Supplement: Supplementary file 3 [file pone.0354353.s004.docx]

**Responses to Reviewers' comments:**

Reviewer's Responses to Questions

**Comments to the Author**

1. Is the manuscript technically sound, and do the data support the conclusions?

The manuscript must describe a technically sound piece of scientific research with data that supports the conclusions. Experiments must have been conducted rigorously, with appropriate controls, replication, and sample sizes. The conclusions must be drawn appropriately based on the data presented.

Reviewer #1: Partly

Many thanks for your comment

2. Has the statistical analysis been performed appropriately and rigorously?

Reviewer #1: No

We will address any specific issue raised in the subsequent sections where the reviewer hiighlights

3. Have the authors made all data underlying the findings in their manuscript fully available?

The [PLOS Data policy](https://eur03.safelinks.protection.outlook.com/?url=http%3A%2F%2Ftrack.editorialmanager.com%2FCL0%2Fhttp%3A%252F%252Fwww.plosone.org%252Fstatic%252Fpolicies.action%2523sharing%2F1%2F010f019a9f2d9d22-367b285c-1d60-4e28-9c53-a210df944de4-000000%2FrY3h9tTpzwJjdeeq18Fj4pb5Q-feaoWo55E7OKk6GE0%3D238&data=05%7C02%7CUche.Anyanwagu%40newcastle.ac.uk%7Cf801437fae8c44095e5808de282fb00d%7C9c5012c9b61644c2a91766814fbe3e87%7C1%7C0%7C638992382659347691%7CUnknown%7CTWFpbGZsb3d8eyJFbXB0eU1hcGkiOnRydWUsIlYiOiIwLjAuMDAwMCIsIlAiOiJXaW4zMiIsIkFOIjoiTWFpbCIsIldUIjoyfQ%3D%3D%7C0%7C%7C%7C&sdata=WR5eDG4gjNoX%2FH%2BnDt07VjszlIJ7l2CL0ypRzQ7Eka4%3D&reserved=0) requires authors to make all data underlying the findings described in their manuscript fully available without restriction, with rare exception (please refer to the Data Availability Statement in the manuscript PDF file). The data should be provided as part of the manuscript or its supporting information, or deposited to a public repository. For example, in addition to summary statistics, the data points behind means, medians and variance measures should be available. If there are restrictions on publicly sharing data—e.g. participant privacy or use of data from a third party—those must be specified.

Reviewer #1: No

We have updated and included the following data availability Statement stated below:

All data used in this study has been requested from SSNAP and the Healthcare Quality Improvement Programme (HQIP) (reference HQIP450) where certain restrictions apply. A data request form must be completed and submitted to SSNAP alongside a Data Access Request form to HQIP for consideration. Requests to access these datasets should be directed to SSNAP, <https://www.strokeaudit.org/Research/Data-requests.aspx>

4. Is the manuscript presented in an intelligible fashion and written in standard English?

PLOS ONE does not copyedit accepted manuscripts, so the language in submitted articles must be clear, correct, and unambiguous. Any typographical or grammatical errors should be corrected at revision, so please note any specific errors here.

Reviewer #1: Yes

Thank you for your comment

5. Review Comments to the Author

Please use the space provided to explain your answers to the questions above. You may also include additional comments for the author, including concerns about dual publication, research ethics, or publication ethics. (Please upload your review as an attachment if it exceeds 20,000 characters)

Reviewer #1: This paper looks at racial/ethnic differences in follow up after stroke, demonstrating worse outcomes despite similar levels of follow up. This is a useful contribution to this neglected aspect of post-acute stroke care. A major strength is the use of a nationally collected prospective database in a country where healthcare is provided free.

Thank you for your comment

Major concerns: The authors present data on the odds of receiving post acute medications, but have not provided the underlying data. In their supplementary tables on medications at baseline, data appears to be missing in a large majority of patients. I recommend the data on follow up medication is provided, including percentage on no apparent treatment and if there is any missing data. I recommend this analysis is restricted to patients with ischaemic stroke for clarity.

Response:

Thank you for pointing this out. The underlying data on the post stroke acute medication is in Supplementary Table 2 (under the subheading of Medications). The reviewer is correct in pointing about the huge percentage of missing data here. This is a limitation with our dataset. Being an ongoing longitudinal dataset, data entry is progressively getting better. We have acknowledged this limitation in the Discussion section (see lines 406 to 411).

Concerning restricting our analysis to only patients with ischaemic stroke, this will exclude an important proportion of those with haemorrhagic stroke (which in our dataset is between 9 to 14%. Moreover, it goes against the aim of our study - which is to assess ethnic differences in post-stroke care and clinical outcomes during the 6 months follow up (irrespective of the type of stroke). As this was an exploratory study it was important to include all stroke patients irrespective of subtype. We would welcome future studies to look at how this may differ in different stroke subtypes.

Outcome data, if available, would be a useful addition e.g. blood pressure measurements at follow up, lipid levels at follow up.

Response:

Thank you for this very important observation. We do not have any data on these outcomes at the 6 months follow up. The dataset only has data on if survivors attended follow up at 6 months or not. Moreover, some of these follow ups occurred via telephone and online and not only face-to-face. SSNAP is a national audit with prespecified categories and questions which we have used in our exploratory study. We agree future studies could look at these specific metrics for further clarity in outcome data but at present we are limited by what the clinical teams are asked to collect as part of SSNAP.

Minor issues:
There is a significant amount of missing/not recorded data. An explicit discussion as to how the authors handled missing data is pertinent.

Response:

Thank you for this very important observation. In real-world clinical data it is not unexpected that there should be a degree of missing data when compared to for example cohort studies. We have pointed this as a limitation of our study in the discussion section. For our main study outcome (ethnicity), due to its sensitive nature, 23.1% of the respondents registered unknown. This can be understood in this context, and it was on this basis that they were coded as a separate category. For other variables like medication use, etc, we coded them as a different category and analysed them as such due to their huge proportion.

We had discussed techniques to look at missing variables in the discussion section as follows:

“Techniques to address missing data e.g. multiple imputation were considered but there were concerns that this would only serve to increase bias in the results as there would be no provide enough justification that the data were missing at random (31). Further, given the size of the dataset it would seem unlikely that spurious associations are being reported (especially if these show effects in a similar direction)”

Further, removing them from the dataset would skew the outcomes of our study. Multiple imputation best serves retrospective cohort studies where some values of the outcomes of interest, in a time-series fashion, are missing. This, again, we felt was inappropriate in this study which uses prospectively collected clinical data where it is not always possible to statistically account for why the data may be missing. Doing this simply amounts to stating (or adding ‘yes’) to those who may never have attended the 6 months review. We feel that this would put a great deal of bias on the credibility of our findings. Studies using the same data (for example <https://pmc.ncbi.nlm.nih.gov/articles/PMC8977818/#R27>) had the same concerns around how to handle missing data which have acknowledged as a limitation due to the nature of the clinical data.

There are systematic differences between the followed up population and those that did not receive follow up. The authors have controlled for this, but I think this could be made clearer in the text of the article.

Response:

Thank you for this. This has been done.

I would suggest the authors point out that in the UK healthcare system, care is free (and for this group of patients, usually medication is also free), particularly as they cite disparities in the US where there is a very different model of healthcare. There have been some papers looking at racial and ethnic differences in the Canadian healthcare system which might assist the discussion of their findings e.g:
Vyas MV, Austin PC, Fang J, Laupadis A, Silver FA, Kapral MK. Immigration status, ethnicity, and long-term outcomes following ischemic stroke. Neurology 2021;96:e1145-e 1155
Vyas MV, Austin PC, Pequeno P, Fang J, Silver FL, Laupacis A, Kapral MK. Incidence of stroke in immigrants to Canada. A province-wide retrospective analysis. Neurology 2021;97:e1192-e1201
Vyas MV et al. Association between immigration status and ambulatory secondary stroke preventive care in Ontario, Canada. Neurology 2024;103:e209536

Thank you to the reviewer for highlighting this important issue. We have stated that the UK is free at the point of use in the introduction. We have also added in a couple of studies from Vyas et al including one of the suggested citations in the discussion. This was to reflect the fact that a similar healthcare system found that there was increased disability seen in immigrants post-stroke and that secondary prevention use in Canada in slightly better in immigrants compared to long term residents. We thank the reviewer for pointing these studies out so that we can reasonably compare findings with similar healthcare systems.

6. PLOS authors have the option to publish the peer review history of their article ([what does this mean?](https://eur03.safelinks.protection.outlook.com/?url=https%3A%2F%2Ftrack.editorialmanager.com%2FCL0%2Fhttps%3A%252F%252Fjournals.plos.org%252Fplosone%252Fs%252Feditorial-and-peer-review-process%2523loc-peer-review-history%2F1%2F010f019a9f2d9d22-367b285c-1d60-4e28-9c53-a210df944de4-000000%2FbD86A94NPYGDjDW-p51cb7WEeVL7mKuA4SNmsXoyIkQ%3D238&data=05%7C02%7CUche.Anyanwagu%40newcastle.ac.uk%7Cf801437fae8c44095e5808de282fb00d%7C9c5012c9b61644c2a91766814fbe3e87%7C1%7C0%7C638992382659364169%7CUnknown%7CTWFpbGZsb3d8eyJFbXB0eU1hcGkiOnRydWUsIlYiOiIwLjAuMDAwMCIsIlAiOiJXaW4zMiIsIkFOIjoiTWFpbCIsIldUIjoyfQ%3D%3D%7C0%7C%7C%7C&sdata=a0sd34kzRIg6B2jYVFfhC3nq0dSa6P13EhskUXCCXBA%3D&reserved=0)). If published, this will include your full peer review and any attached files.

If you choose “no”, your identity will remain anonymous but your review may still be made public.

**Do you want your identity to be public for this peer review?** For information about this choice, including consent withdrawal, please see our [Privacy Policy](https://eur03.safelinks.protection.outlook.com/?url=https%3A%2F%2Ftrack.editorialmanager.com%2FCL0%2Fhttps%3A%252F%252Fwww.plos.org%252Fprivacy-policy%2F1%2F010f019a9f2d9d22-367b285c-1d60-4e28-9c53-a210df944de4-000000%2F7jzRkDPuWTuMisXidS7mtKM1KOgmxXeRRFexw2slff8%3D238&data=05%7C02%7CUche.Anyanwagu%40newcastle.ac.uk%7Cf801437fae8c44095e5808de282fb00d%7C9c5012c9b61644c2a91766814fbe3e87%7C1%7C0%7C638992382659381746%7CUnknown%7CTWFpbGZsb3d8eyJFbXB0eU1hcGkiOnRydWUsIlYiOiIwLjAuMDAwMCIsIlAiOiJXaW4zMiIsIkFOIjoiTWFpbCIsIldUIjoyfQ%3D%3D%7C0%7C%7C%7C&sdata=qnMSm0LGxIxHYaciQaVahPNkOIoY%2Fkd4Ph%2FwvICbPBY%3D&reserved=0).

Reviewer #1: **Yes:** Dennis Briley

[NOTE: If reviewer comments were submitted as an attachment file, they will be attached to this email and accessible via the submission site. Please log into your account, locate the manuscript record, and check for the action link "View Attachments". If this link does not appear, there are no attachment files.]

To ensure your figures meet our technical requirements, please review our figure guidelines: <https://journals.plos.org/plosone/s/figures>

You may also use PLOS’s free figure tool, NAAS, to help you prepare publication quality figures: <https://journals.plos.org/plosone/s/figures#loc-tools-for-figure-preparation>.

NAAS will assess whether your figures meet our technical requirements by comparing each figure against our figure specifications.

*In compliance with data protection regulations, you may request that we remove your personal registration details at any time.* [*(Remove my information/details)*](https://eur03.safelinks.protection.outlook.com/?url=https%3A%2F%2Ftrack.editorialmanager.com%2FCL0%2Fhttps%3A%252F%252Fwww.editorialmanager.com%252Fpone%252Flogin.asp%253Fa%3Dr%2F1%2F010f019a9f2d9d22-367b285c-1d60-4e28-9c53-a210df944de4-000000%2FHWnAzjYG9qVyJsOuGCroHoZex_15S8xmZJVmJS5yi0w%3D238&data=05%7C02%7CUche.Anyanwagu%40newcastle.ac.uk%7Cf801437fae8c44095e5808de282fb00d%7C9c5012c9b61644c2a91766814fbe3e87%7C1%7C0%7C638992382659399441%7CUnknown%7CTWFpbGZsb3d8eyJFbXB0eU1hcGkiOnRydWUsIlYiOiIwLjAuMDAwMCIsIlAiOiJXaW4zMiIsIkFOIjoiTWFpbCIsIldUIjoyfQ%3D%3D%7C0%7C%7C%7C&sdata=1PFTuI2dJlHluS6MrC5BBGbE7LEKDibgqLjF4V8uoq8%3D&reserved=0)*. Please contact the publication office if you have any questions.*
