# Supplementary material for: Ethnic disparities in the longer-term clinical care and outcomes of stroke patients in England
Source: PLoS One. 2026 Aug 5;21(8):e0354353. doi: 10.1371/journal.pone.0354353 (PMC13440867; doi:10.1371/journal.pone.0354353)
Supplement: Supplementary file 4 [file pone.0354353.s005.docx]

Dear editorial team and Dr Briley,

First of all please accept my sincerest thanks for another really comprehensive review. The suggestions were incredibly help and has shaped the paper to be much stronger and clearer. Where there have been some concerns outlined by Dr. Briley we have highlighted this as a limitation of our study to prevent over-generalisation. We hope that this paper is now suitable for peer-reviewed publication

Yours sincerely

Dr. Eugene Tang

**Reviewer #1:** The authors present data on the odds of receiving post acute medications (Table 3), which I commented on previously. I apologize if I am missing something, but I cannot find the supporting data in the supplementary tables. The data I do see implies 84% of the information is "missing" and I see no data regarding lipid therapy in any of the tables. I think either the underlying data to support this table should be included. If indeed this table is based on missing data in 84% of subjects, then I suggest it and the supporting text is removed.

**Thank you for highlighting this and please accept our apologies for the confusion. The lipid therapy odds ratio can be found as the last column of table 3 on page 11 along with all the other post-acute medication data. In it you will find the data for the patients who have attended their 6 month review and the medication associated with their review. With regards to the missing data, figure 1 outlines that we have 41,980 patients with missing follow up data and therefore the data presented in table 3 are the ones which have the outcome data we have outlined. The medications section of both supplementary 1 and 2 do not refer to the 6 month post-acute medication taking but rather whether on admission the patients were taking this prior to their stroke. This is therefore not the data in relation to post-acute medications which are all highlighted in table 3. We hope this clarifies this point.**

I would again recommend this table is based purely on ischaemic stroke. To include all stroke will introduce confounding by indication rather than the differences being attributable to ethnic or racial differences.

**We thank the reviewer again for pointing this out. In post-acute services, there is no differentiation between the type of stroke and subsequent clinical management. For example a patient having had an ischaemic stroke would be seen in the same post-acute setting i.e. 4-6 week and 6 month review clinic as those with haemorrhagic stroke and the desired outcomes would be the same as dictated by SSNAP. Our study is exploratory and focusses on what we can do to improve clinical services for all stroke-survivors irrespective of underlying cause. We do recognise this point that the reviewer has made and we have put it in as a limitation of our study and also scope for future work.**

I have some general style comments for the authors. There is variability in discussing the racial/ethnic groups. It might be easier to say “non-White” rather than the clumsy “other than White”. I advise the authors use consistent phrasing through the paper.

**Thank you, we have changed this to be consistently “non-White” as advised.**

Secondly, there is a lot of use of “however”, “on the other hand” etc which could be removed and would make the paper more readable.

**Thank you, we have taken this suggestion and amended**

There was no comment about COVID in the discussion (even if to say “we found no significant change in follow up assessments with the COVID outbreak”)

**Thank you, we have added in a sentence to reflect this at the beginning of the discussion**

Some specific comments/suggestions:


Under “Baseline characteristics Line 170. Perhaps say “ As anticipated, the highest proportion of strokes was in the elderly with the age group 80-84 showing the highest proportion (16.2%)”

**We have changed the sentence as suggested**

Under 6th Month review line 222 I suggest restructuring the paragraph and it might be appropriate to replace the word “need” (eg the provision of cognitive support was similar....)

**Thank you for his suggestion. The outcome was less about provision and more about identification. As the dataset is capturing whether the individual was identified as requiring cognitive support we have changed the sentence to reflect this more clearly.**

eg “The need for psychological support and the odds of change of residence of abode in all ethnic groups compared to White patients is shown in table 2. After adjustment for age and stroke severity, the need for cognitive support was similar in Black and White patients, non-significantly lower in the mixed and other ethnic groups but was 19% in Asian patients and 24% higher in the unknown ethnic groups”

**We thank the reviewer for this suggestion which we have taken on board and amended the manuscript accordingly.**

I think the Discussion would benefit from a major re-edit.
1st sentence line 262 is very cumbersome and the authors may wish to consider something along the lines of:

“Our findings demonstrate despite non-White stroke survivors having the
highest attendance rate for 6-month follow-up, there were disparities in health
outcomes. Post-stroke comorbidities were more likely in all other ethnic groups
combined, in particular recurrent stroke, and hospital readmissions for other
illnesses in Black and Asian stroke-survivors. The odds of having a myocardial infarction within six months post-stroke was also significantly higher in Asian patients. Conversely, atrial fibrillation pre- and post-stroke was less likely in non-White stroke-survivors. There were differences in use of secondary prevention. Black and Asian stroke-survivors
were more likely to be taking antihypertensives, which may reflect the fact that a higher
proportion had hypertension pre-stroke. However, all other ethnic groups combined stroke
survivors are less likely to be taking lipid therapy. The odds of antiplatelet use were
higher in Asian patients than in other ethnicities and anticoagulant use was lower in non-White patients, probably reflecting the lower prevalence of atrial fibrillation in non-Whites. There was a similar need for cognitive support in Black and Mixed stroke-survivors compared to White patients with Asian patients needing more psychological support”

**Thank you, we have taken this suggestion and amended**

I don’t think the word need is correct- would not “use of” be better?

**Thank you for allowing us to clarify. Please refer to our point above as to what the SSNAP dataset asks for which is around identification of those who need psychological support rather than its use.**

The text of the third paragraph beginning with line 289 is difficult to follow and the sentence on line 297 makes no sense to me. I suggest clarifying the text e.g.

“Attendance to the 6-months follow up review is generally low (26.2%), which is slightly lower than national data [13]. Although non-White patients were more likely to attend their 6-month review, a greater proportion of Black patients, Asian patients, and other ethnic groups were reviewed via telephone rather than in person. For those who attended their 6-month reviews, non-White patients were more likely to have comorbidities at baseline including hypertension and diabetes. This may be a factor in the higher recurrent stroke rate. Previous studies have found that Black patients, particularly at younger ages, were more
strongly linked with both earlier acute onset [14] and recurrence [15] and may not be fully
attenuated by traditional stroke risk factor management [5]. This difference was not confined
to recurrent stroke as both hospitalisation for other illnesses and myocardial infarction at 6-
month follow-up were higher in all other ethnic groups combined patients in our study. This
suggests that there may be benefits to earlier or more targeted follow-up for these at-risk
populations. Non-attenders for 6-month reviews tended to be younger in non-White patients (75-85 years age group among White patients, 55-64 years in Black patients; 65- 69 years in Asian patients and 60-64 years mixed ethnic group), and were more likely to have risk
factors for recurrent stroke such as hypertension in other than White stroke-survivors would appear to be a group that should be particularly targeted. Previous studies comparing immigrant health versus long-term residents, immigrants (mainly from Asian ethnicities) had a higher risk of being disabled following a stroke on discharge compared to long-term residents in a comparable healthcare system to the UK [16]. Future research should look at where in the stroke care pathway would be best to intervene to reduce the impact of stroke for those from minority ethnic populations”

**Thank you, we have taken this suggestion and amended**

I recommend removing the clause “although it should be noted that in general more immigrants were tested for hyperlipidaemia” in line 336. I can’t see relevance of this unless there is data to indicate less lipid testing in stroke in UK

**Thank you, we have taken this suggestion and amended**

Line 338 perhaps change to “may also reduce adverse cognitive deficits”

**Thank you, we have taken this suggestion and amended**

The last sentence of this paragraph (line 348) seems redundant and could be removed.

**Thank you, we have taken this suggestion and amended**

I suggest restructuring the paragraph on post stroke cognitive issues (paragraph starting line 351). I think caution is needed in interpretation of findings as no direct outcome measure of cognitive function has been provided, just the indirect one of use of services, which may have other explanations. The authors might consider rewriting e.g.:

Cognitive impairment is common post-stroke and also associated with ethnicity [24]. Neuropsychiatric outcomes such as depression, anxiety, fatigue, and apathy are common post-stroke [25]. Depression post-stroke has an estimated pooled prevalence of around a third (27%) from observational studies. Those experiencing depression within 3 months post-stroke are at risk of remaining depressed a year after stroke [26]. Similarly, post-stroke anxiety can occur in 1 in 4 stroke-survivors [27]. Psychological outcomes such as depression and anxiety are often linked to cognitive deficits. Links between ethnicity and post-stroke outcomes such as depression are not consistent [28] with some finding no differences [29]. There do seem to be some ethnic differences when it comes to cognition with more cognitive impairment suggestive of dementia found in non-Europeans compared with Europeans [30]. Post stroke cognitive impairment is more common in those of Caribbean/African ethnicities [24]. In our study, at follow up, the use of psychological support was higher in Asian patients compared to White patients. Conversely, those from Mixed or Black ethnicities had similar levels to Whites in terms of receiving cognitive support. It is known that post-acute access to clinical psychology is often limited and below recommendations [31]. The degree of utilization of cognitive support offered to Black patients may be more of a reflection of the available clinical services or to cultural understanding of what normal or abnormal cognition may entail. Clinical services need to find ways to not only identify these individuals requiring psychological and/or cognitive support but also ways of following them up to ensure earlier detection of common post-stroke outcomes such as depression, anxiety, and cognitive impairment.

**Thank you, we have taken this suggestion and amended**

The paragraph beginning with Line 374 is confusing. Do the authors mean their study s representative of the nationally acquired data of SSNAP? The sentence in line 379 is unclear (The low 6-month follow-up data could be indicative of a poor reflection of the post-acute stroke population) and seems to be mean the patients are being blamed for non-attendance? I recommend this paragraph is rewritten for clarity.

**Thank you for allowing us to clarify. Line 374 refers to the fact that the ethic profile of our sample is similar to the general UK population. The sentence was more to illustrate the strengths of the study. We have simplified it to say that it is representative of the national ethnicity profile and then led into our limitations.**

**With regards to line 379 this was more of a reflection of the clinical teams data entry rather than the patients themselves. We have amended this for clarification.**
