# Supplementary material for: Ethnic disparities in the longer-term clinical care and outcomes of stroke patients in England
Source: PLoS One. 2026 Aug 5;21(8):e0354353. doi: 10.1371/journal.pone.0354353 (PMC13440867; doi:10.1371/journal.pone.0354353)
Supplement: Supplementary file 5 [file pone.0354353.s006.docx]

**4. Have the authors made all data underlying the findings in their manuscript fully available?**

The [PLOS Data policy](https://eur03.safelinks.protection.outlook.com/?url=http%3A%2F%2Ftrack.editorialmanager.com%2FCL0%2Fhttp%3A%252F%252Fwww.plosone.org%252Fstatic%252Fpolicies.action%2523sharing%2F1%2F010f019c6492444d-a3e05a6f-34f3-493d-af4a-19914c95b84f-000000%2F9kfiJAd18MIJXUqXzeTHDJI-dbkCbKXZbFQZ-csNcUw%3D250&data=05%7C02%7CEugene.Tang%40newcastle.ac.uk%7C968a475196cf4a86ee6d08ded2cb071c%7C9c5012c9b61644c2a91766814fbe3e87%7C1%7C0%7C639179966626146592%7CUnknown%7CTWFpbGZsb3d8eyJFbXB0eU1hcGkiOnRydWUsIlYiOiIwLjAuMDAwMCIsIlAiOiJXaW4zMiIsIkFOIjoiTWFpbCIsIldUIjoyfQ%3D%3D%7C0%7C%7C%7C&sdata=vBBkQAf1taejgweDnwNSSnZrbKx5%2BNnkjonGAD4YO5Y%3D&reserved=0) requires authors to make all data underlying the findings described in their manuscript fully available without restriction, with rare exception (please refer to the Data Availability Statement in the manuscript PDF file). The data should be provided as part of the manuscript or its supporting information, or deposited to a public repository. For example, in addition to summary statistics, the data points behind means, medians and variance measures should be available. If there are restrictions on publicly sharing data—e.g. participant privacy or use of data from a third party—those must be specified.

Reviewer #1: No

Please do note that the data underlying this study is available from SSNAP and HQIP as outlined in the paper. The data is from a national audit of stroke patients and their care. The aggregated data is available publicly via SSNAP’s website but this study was conducted at patient-level data. This ultimately means that additional permissions and requirements are needed as outlined in the manuscript and approval was achieved. This is made clear in the data availability statement.

**6. Review Comments to the Author**

**Please use the space provided to explain your answers to the questions above. You may also include additional comments for the author, including concerns about dual publication, research ethics, or publication ethics. (Please upload your review as an attachment if it exceeds 20,000 characters)**

Reviewer #1: My major concern continues to be the lack of supporting data for table 3. Table 3 purely shows the odds ratio. As a ridiculous example, it cannot be discerned if that means one white patient was taking an antiplatelet agent, one black person an antiplatelet agent for 173 days....based on the missing data listed in the supplementary tables for medication use, this would seem to be the logical conclusion. Table 1 which also presents odds ratio does provide the source data i.e. the absolute number of patients and percentages. With the large amount of missing data listed for medications listed in the supplementary tables on admission, the readers will need to know what degree of missingness the 6 month medication data is based on. The data for calculating the OR in table 2 should also be provided. This could be easily done by adding these data to supplementary table 2. I note that it is PLOS One policy that the data should be available: "The PLOS Data policy requires authors to make all data underlying the findings described in their manuscript fully available without restriction, with rare exception (please refer to the Data Availability Statement in the manuscript PDF file). The data should be provided as part of the manuscript or its supporting information, or deposited to a public repository. For example, in addition to summary statistics, the data points behind means, medians and variance measures should be available."

Thank you for allowing us to clarify this point. In supplementary table 2 you will find that this reflects the data of the patients who attended their 6 month reviews in terms of the hospital admission. The medications that the reviewer refers to are the antiplatelets, anticoagulants and the type. These are the medications that the patients were taking PRIOR to their stroke. This has no bearing on whether or not they have subsequently taken their secondary prevention medication as outlined in table 3. We do take the point though that given the antiplatelets and anticoagulants are an outcome of the 6 month review that their inclusion with the degree of missingness would be an issue and it would be difficult to gain a meaningful conclusion on this aspect of secondary prevention. We have removed these two aspects and just focussed on antihypertensives and lipid therapy. We have also highlighted this as a limitation of the study and hope this is now acceptable.

With regards to table 2, we are somewhat confused by the reviewers comments. The absolute number and percentages are already included in the table. All data for this study have already been included in either the main manuscript or the supplementary tables. Given the nature of this clinical data, any requests need to be made to HQIP and SSNAP as outlined in the data availability statement.

I again recommend that the data on medication use at the 6 month visit be purely for the ischaemic stroke patients. Historically, before imaging, stroke included both cerebral haemorrhage and ischaemic stroke, but they are not the same diseases and do not have the same need for medication, for example one would not treat a patient with cerebral haemorrhage with anticoagulation. To then conclude as the authors do in their abstract that “Black and Asian stroke-survivors experience worse outcomes and lower use of secondary prevention despite similar clinic attendance” when their data actually show different disease preponderance in different ethnic groups which are treated differently, is wrong. In my opinion the lumping together of ischaemic stroke and cerebral haemorrhage as one disease is not acceptable for this aspect of their work.

We thank the reviewer for further emphasising this point. We do take the point around anticoagulation for example and have removed this from table 3 as explained above. We would like to emphasise several points to further reassure the reviewer/editorial team. As per national guidance ([https://www.strokeguideline.org/chapter/long-term-management-and-secondary-prevention](https://www.strokeguideline.org/chapter/long-term-management-and-secondary-prevention/?_gl=1*19l3edj*_up*MQ..*_ga*MjAwODg0MTM1My4xNzgyNDY2MzUz*_ga_EE3BZMVLRT*czE3ODI0NjYzNTIkbzEkZzEkdDE3ODI0NjYzNTUkajU3JGwwJGgw#457)) there is no difference in terms of secondary prevention (blood pressure and lipid therapy) for ischaemic or haemorrhagic stroke. Our conclusions can therefore be justified in this context following removal of the antiplatelet and anticoagulant data. Further, this specific detail was not the main aim of this study particularly when the same services in the UK look after both groups along the same care pathway. This is about care standards rather than a mechanistic paper as it would be underpowered to get the level of detail needed to draw out such conclusions. We set out to look for racial disparities in care rather than the granularity requested by the reviewer. We have emphasised that this study is exploratory and serves as a pointer to other studies who wish to explore some of these additional areas in detail. Our exploratory analysis is about the meta question of ethnicity and achieving care standards rather than a mechanistic question about ethnicity causing differential effects in care. We have emphasied this in the limitations section. Our findings are in no way an anomaly in the wider literature but serves to spotlight the specific areas that clinical teams could focus on in both clinical practice and in research. Finally, we would like to emphasise that the data used in SSNAP is a clinical audit that requires the clinician to input the data in real time. It is different to other cohort or registry datasets and we have emphasised the type of dataset used. We hope this has now sufficiently answered this reviewer’s query.
