# Supplementary material for: Ethnic disparities in the longer-term clinical care and outcomes of stroke patients in England
Source: PLoS One. 2026 Aug 5;21(8):e0354353. doi: 10.1371/journal.pone.0354353 (PMC13440867; doi:10.1371/journal.pone.0354353)
Supplement: Peer review history

# Peer review: Decision Letter 0

**DOI:** 10.1371/journal.pone.0354353.r001

**Authors:** Antony Bayer

19 Nov 2025

PONE-D-25-38926

Ethnic Disparities in the Longer-Term Clinical Care and Outcomes of Stroke Patients in England

PLOS ONE

Dear Dr. Tang,

Thank you for submitting your manuscript to PLOS ONE. After careful consideration, we feel that it has merit but does not fully meet PLOS ONE’s publication criteria as it currently stands. Therefore, we invite you to submit a revised version of the manuscript that addresses the points raised during the review process. In particular, the reviewer highlights issues around data on medications and the amount of missing data.

Please submit your revised manuscript by Jan 03 2026 11:59PM. If you will need more time than this to complete your revisions, please reply to this message or contact the journal office at plosone@plos.org. When you're ready to submit your revision, log on to https://www.editorialmanager.com/pone/ and select the 'Submissions Needing Revision' folder to locate your manuscript file.

Please include the following items when submitting your revised manuscript:

- A rebuttal letter that responds to each point raised by the academic editor and reviewer(s). You should upload this letter as a separate file labeled 'Response to Reviewers'.
- A marked-up copy of your manuscript that highlights changes made to the original version. You should upload this as a separate file labeled 'Revised Manuscript with Track Changes'.
- An unmarked version of your revised paper without tracked changes. You should upload this as a separate file labeled 'Manuscript'.

If you would like to make changes to your financial disclosure, please include your updated statement in your cover letter. Guidelines for resubmitting your figure files are available below the reviewer comments at the end of this letter.

If applicable, we recommend that you deposit your laboratory protocols in protocols.io to enhance the reproducibility of your results. Protocols.io assigns your protocol its own identifier (DOI) so that it can be cited independently in the future. For instructions see: https://journals.plos.org/plosone/s/submission-guidelines#loc-laboratory-protocols. Additionally, PLOS ONE offers an option for publishing peer-reviewed Lab Protocol articles, which describe protocols hosted on protocols.io. Read more information on sharing protocols at https://plos.org/protocols?utm_medium=editorial-email&utm_source=authorletters&utm_campaign=protocols.

We look forward to receiving your revised manuscript.

Kind regards,

Antony Bayer

Academic Editor

PLOS ONE

Journal requirements:

When submitting your revision, we need you to address these additional requirements.

1. Please ensure that your manuscript meets PLOS ONE's style requirements, including those for file naming. The PLOS ONE style templates can be found at

https://journals.plos.org/plosone/s/file?id=wjVg/PLOSOne_formatting_sample_main_body.pdf and

https://journals.plos.org/plosone/s/file?id=ba62/PLOSOne_formatting_sample_title_authors_affiliations.pdf

2. Please note that your Data Availability Statement is currently missing [the repository name and/or the DOI/accession number of each dataset OR a direct link to access each database]. If your manuscript is accepted for publication, you will be asked to provide these details on a very short timeline. We therefore suggest that you provide this information now, though we will not hold up the peer review process if you are unable.

3. Your ethics statement should only appear in the Methods section of your manuscript. If your ethics statement is written in any section besides the Methods, please move it to the Methods section and delete it from any other section. Please ensure that your ethics statement is included in your manuscript, as the ethics statement entered into the online submission form will not be published alongside your manuscript.

4. If the reviewer comments include a recommendation to cite specific previously published works, please review and evaluate these publications to determine whether they are relevant and should be cited. There is no requirement to cite these works unless the editor has indicated otherwise.

Additional Editor Comments (if provided):

[Note: HTML markup is below. Please do not edit.]

Reviewers' comments:

Reviewer's Responses to Questions

**Comments to the Author**

1. Is the manuscript technically sound, and do the data support the conclusions?

The manuscript must describe a technically sound piece of scientific research with data that supports the conclusions. Experiments must have been conducted rigorously, with appropriate controls, replication, and sample sizes. The conclusions must be drawn appropriately based on the data presented.

Reviewer #1: Partly

**********

2. Has the statistical analysis been performed appropriately and rigorously?

Reviewer #1: No

**********

3. Have the authors made all data underlying the findings in their manuscript fully available?

The PLOS Data policy requires authors to make all data underlying the findings described in their manuscript fully available without restriction, with rare exception (please refer to the Data Availability Statement in the manuscript PDF file). The data should be provided as part of the manuscript or its supporting information, or deposited to a public repository. For example, in addition to summary statistics, the data points behind means, medians and variance measures should be available. If there are restrictions on publicly sharing data—e.g. participant privacy or use of data from a third party—those must be specified.

Reviewer #1: No

**********

4. Is the manuscript presented in an intelligible fashion and written in standard English?

PLOS ONE does not copyedit accepted manuscripts, so the language in submitted articles must be clear, correct, and unambiguous. Any typographical or grammatical errors should be corrected at revision, so please note any specific errors here.

Reviewer #1: Yes

**********

5. Review Comments to the Author

Please use the space provided to explain your answers to the questions above. You may also include additional comments for the author, including concerns about dual publication, research ethics, or publication ethics. (Please upload your review as an attachment if it exceeds 20,000 characters)

Reviewer #1: This paper looks at racial/ethnic differences in follow up after stroke, demonstrating worse outcomes despite similar levels of follow up. This is a useful contribution to this neglected aspect of post-acute stroke care. A major strength is the use of a nationally collected prospective database in a country where healthcare is provided free.

Major concerns: The authors present data on the odds of receiving post acute medications, but have not provided the underlying data. In their supplementary tables on medications at baseline, data appears to be missing in a large majority of patients. I recommend the data on follow up medication is provided, including percentage on no apparent treatment and if there is any missing data. I recommend this analysis is restricted to patients with ischaemic stroke for clarity.

Outcome data, if available, would be a useful addition e.g. blood pressure measurements at follow up, lipid levels at follow up.

Minor issues:

There is a significant amount of missing/not recorded data. An explicit discussion as to how the authors handled missing data is pertinent.

There are systematic differences between the followed up population and those that did not receive follow up. The authors have controlled for this, but I think this could be made clearer in the text of the article.

I would suggest the authors point out that in the UK healthcare system, care is free (and for this group of patients, usually medication is also free), particularly as they cite disparities in the US where there is a very different model of healthcare. There have been some papers looking at racial and ethnic differences in the Canadian healthcare system which might assist the discussion of their findings e.g:

Vyas MV, Austin PC, Fang J, Laupadis A, Silver FA, Kapral MK. Immigration status, ethnicity, and long-term outcomes following ischemic stroke. Neurology 2021;96:e1145-e 1155

Vyas MV, Austin PC, Pequeno P, Fang J, Silver FL, Laupacis A, Kapral MK. Incidence of stroke in immigrants to Canada. A province-wide retrospective analysis. Neurology 2021;97:e1192-e1201

Vyas MV et al. Association between immigration status and ambulatory secondary stroke preventive care in Ontario, Canada. Neurology 2024;103:e209536

**********

6. PLOS authors have the option to publish the peer review history of their article (what does this mean?). If published, this will include your full peer review and any attached files.

If you choose “no”, your identity will remain anonymous but your review may still be made public.

**Do you want your identity to be public for this peer review?**  For information about this choice, including consent withdrawal, please see our Privacy Policy.

Reviewer #1: **Yes:** Dennis Briley

**********

[NOTE: If reviewer comments were submitted as an attachment file, they will be attached to this email and accessible via the submission site. Please log into your account, locate the manuscript record, and check for the action link "View Attachments". If this link does not appear, there are no attachment files.]

To ensure your figures meet our technical requirements, please review our figure guidelines: https://journals.plos.org/plosone/s/figures

You may also use PLOS’s free figure tool, NAAS, to help you prepare publication quality figures: https://journals.plos.org/plosone/s/figures#loc-tools-for-figure-preparation.

NAAS will assess whether your figures meet our technical requirements by comparing each figure against our figure specifications.

# Author comment: Author response to Decision Letter 1

**DOI:** 10.1371/journal.pone.0354353.r002

11 Dec 2025

We thank the reviewer for his expertise. Please see our attached rebuttal

Attachment Submitted filename: Responses to Reviewers.docx

# Peer review: Decision Letter 1

**DOI:** 10.1371/journal.pone.0354353.r003

**Authors:** Antony Bayer

2 Jan 2026

PONE-D-25-38926R1

Ethnic Disparities in the Longer-Term Clinical Care and Outcomes of Stroke Patients in England

PLOS One

Dear Dr. Tang,

Thank you for submitting your manuscript to PLOS ONE and for your attention to the previous comments of the reviewer. **You will see below that he has some outstanding concerns. Given the very large amount of missing data,  I would agree that you should consider if Table 3 and associated text might best be deleted. The writing style would also benefit from thorough review, to aid readability and ensure clarity, using simple but clear terminology (e.g. 'Non-white" rather than "ethnic minority (excluding white minorities)"). The reviewer has made some very helpful suggestions.**  After careful consideration, therefore, we feel that your paper has merit but does not fully meet PLOS ONE’s publication criteria as it currently stands. We invite you to submit a revised version of the manuscript that addresses the points raised during the review process.

Please submit your revised manuscript by Feb 16 2026 11:59PM. If you will need more time than this to complete your revisions, please reply to this message or contact the journal office at plosone@plos.org. When you're ready to submit your revision, log on to https://www.editorialmanager.com/pone/ and select the 'Submissions Needing Revision' folder to locate your manuscript file.

Please include the following items when submitting your revised manuscript:

- A letter that responds to each point raised by the academic editor and reviewer(s). You should upload this letter as a separate file labeled 'Response to Reviewers'.
- A marked-up copy of your manuscript that highlights changes made to the original version. You should upload this as a separate file labeled 'Revised Manuscript with Track Changes'.
- An unmarked version of your revised paper without tracked changes. You should upload this as a separate file labeled 'Manuscript'.

If you would like to make changes to your financial disclosure, please include your updated statement in your cover letter. Guidelines for resubmitting your figure files are available below the reviewer comments at the end of this letter.

If applicable, we recommend that you deposit your laboratory protocols in protocols.io to enhance the reproducibility of your results. Protocols.io assigns your protocol its own identifier (DOI) so that it can be cited independently in the future. For instructions see: https://journals.plos.org/plosone/s/submission-guidelines#loc-laboratory-protocols. Additionally, PLOS ONE offers an option for publishing peer-reviewed Lab Protocol articles, which describe protocols hosted on protocols.io. Read more information on sharing protocols at https://plos.org/protocols?utm_medium=editorial-email&utm_source=authorletters&utm_campaign=protocols.

We look forward to receiving your revised manuscript.

Kind regards,

Antony Bayer

Academic Editor

PLOS One

**Journal Requirements:**

If the reviewer comments include a recommendation to cite specific previously published works, please review and evaluate these publications to determine whether they are relevant and should be cited. There is no requirement to cite these works unless the editor has indicated otherwise.

[Note: HTML markup is below. Please do not edit.]

Reviewers' comments:

Reviewer's Responses to Questions

**Comments to the Author**

1. If the authors have adequately addressed your comments raised in a previous round of review and you feel that this manuscript is now acceptable for publication, you may indicate that here to bypass the “Comments to the Author” section, enter your conflict of interest statement in the “Confidential to Editor” section, and submit your "Accept" recommendation.

Reviewer #1: (No Response)

**********

2. Is the manuscript technically sound, and do the data support the conclusions?

The manuscript must describe a technically sound piece of scientific research with data that supports the conclusions. Experiments must have been conducted rigorously, with appropriate controls, replication, and sample sizes. The conclusions must be drawn appropriately based on the data presented.

Reviewer #1: Partly

**********

3. Has the statistical analysis been performed appropriately and rigorously?

Reviewer #1: Yes

**********

4. Have the authors made all data underlying the findings in their manuscript fully available?

The PLOS Data policy requires authors to make all data underlying the findings described in their manuscript fully available without restriction, with rare exception (please refer to the Data Availability Statement in the manuscript PDF file). The data should be provided as part of the manuscript or its supporting information, or deposited to a public repository. For example, in addition to summary statistics, the data points behind means, medians and variance measures should be available. If there are restrictions on publicly sharing data—e.g. participant privacy or use of data from a third party—those must be specified.

Reviewer #1: No

**********

5. Is the manuscript presented in an intelligible fashion and written in standard English?

PLOS ONE does not copyedit accepted manuscripts, so the language in submitted articles must be clear, correct, and unambiguous. Any typographical or grammatical errors should be corrected at revision, so please note any specific errors here.

Reviewer #1: No

**********

6. Review Comments to the Author

Please use the space provided to explain your answers to the questions above. You may also include additional comments for the author, including concerns about dual publication, research ethics, or publication ethics. (Please upload your review as an attachment if it exceeds 20,000 characters)

**Reviewer #1:** The authors present data on the odds of receiving post acute medications (Table 3), which I commented on previously. I apologize if I am missing something, but I cannot find the supporting data in the supplementary tables. The data I do see implies 84% of the information is "missing" and I see no data regarding lipid therapy in any of the tables. I think either the underlying data to support this table should be included. If indeed this table is based on missing data in 84% of subjects, then I suggest it and the supporting text is removed. I would again recommend this table is based purely on ischaemic stroke. To include all stroke will introduce confounding by indication rather than the differences being attributable to ethnic or racial differences.

I have some general style comments for the authors. There is variability in discussing the racial/ethnic groups. It might be easier to say “non-White” rather than the clumsy “other than White”. I advise the authors use consistent phrasing through the paper. Secondly, there is a lot of use of “however”, “on the other hand” etc which could be removed and would make the paper more readable.

There was no comment about COVID in the discussion (even if to say “we found no significant change in follow up assessments with the COVID outbreak”)

Some specific comments/suggestions:

Under “Baseline characteristics Line 170. Perhaps say “ As anticipated, the highest proportion of strokes was in the elderly with the age group 80-84 showing the highest proportion (16.2%)”

Under 6th Month review line 222 I suggest restructuring the paragraph and it might be appropriate to replace the word “need” (eg the provision of cognitive support was similar....)

eg “The need for psychological support and the odds of change of residence of abode in all ethnic groups compared to White patients is shown in table 2. After adjustment for age and stroke severity, the need for cognitive support was similar in Black and White patients, non-significantly lower in the mixed and other ethnic groups but was 19% in Asian patients and 24% higher in the unknown ethnic groups”

I think the Discussion would benefit from a major re-edit.

1st sentence line 262 is very cumbersome and the authors may wish to consider something along the lines of:

“Our findings demonstrate despite non-White stroke survivors having the

highest attendance rate for 6-month follow-up, there were disparities in health

outcomes. Post-stroke comorbidities were more likely in all other ethnic groups

combined, in particular recurrent stroke, and hospital readmissions for other

illnesses in Black and Asian stroke-survivors. The odds of having a myocardial infarction within six months post-stroke was also significantly higher in Asian patients. Conversely, atrial fibrillation pre- and post-stroke was less likely in non-White stroke-survivors. There were differences in use of secondary prevention. Black and Asian stroke-survivors

were more likely to be taking antihypertensives, which may reflect the fact that a higher

proportion had hypertension pre-stroke. However, all other ethnic groups combined stroke

survivors are less likely to be taking lipid therapy. The odds of antiplatelet use were

higher in Asian patients than in other ethnicities and anticoagulant use was lower in non-White patients, probably reflecting the lower prevalence of atrial fibrillation in non-Whites. There was a similar need for cognitive support in Black and Mixed stroke-survivors compared to White patients with Asian patients needing more psychological support”

I don’t think the word need is correct- would not “use of” be better?

The text of the third paragraph beginning with line 289 is difficult to follow and the sentence on line 297 makes no sense to me. I suggest clarifying the text e.g.

“Attendance to the 6-months follow up review is generally low (26.2%), which is slightly lower than national data [13]. Although non-White patients were more likely to attend their 6-month review, a greater proportion of Black patients, Asian patients, and other ethnic groups were reviewed via telephone rather than in person. For those who attended their 6-month reviews, non-White patients were more likely to have comorbidities at baseline including hypertension and diabetes. This may be a factor in the higher recurrent stroke rate. Previous studies have found that Black patients, particularly at younger ages, were more

strongly linked with both earlier acute onset [14] and recurrence [15] and may not be fully

attenuated by traditional stroke risk factor management [5]. This difference was not confined

to recurrent stroke as both hospitalisation for other illnesses and myocardial infarction at 6-

month follow-up were higher in all other ethnic groups combined patients in our study. This

suggests that there may be benefits to earlier or more targeted follow-up for these at-risk

populations. Non-attenders for 6-month reviews tended to be younger in non-White patients (75-85 years age group among White patients, 55-64 years in Black patients; 65- 69 years in Asian patients and 60-64 years mixed ethnic group), and were more likely to have risk

factors for recurrent stroke such as hypertension in other than White stroke-survivors would appear to be a group that should be particularly targeted. Previous studies comparing immigrant health versus long-term residents, immigrants (mainly from Asian ethnicities) had a higher risk of being disabled following a stroke on discharge compared to long-term residents in a comparable healthcare system to the UK [16]. Future research should look at where in the stroke care pathway would be best to intervene to reduce the impact of stroke for those from minority ethnic populations”

I recommend removing the clause “although it should be noted that in general more immigrants were tested for hyperlipidaemia” in line 336. I can’t see relevance of this unless there is data to indicate less lipid testing in stroke in UK

Line 338 perhaps change to “may also reduce adverse cognitive deficits”

The last sentence of this paragraph (line 348) seems redundant and could be removed.

I suggest restructuring the paragraph on post stroke cognitive issues (paragraph starting line 351). I think caution is needed in interpretation of findings as no direct outcome measure of cognitive function has been provided, just the indirect one of use of services, which may have other explanations. The authors might consider rewriting e.g.:

Cognitive impairment is common post-stroke and also associated with ethnicity [24]. Neuropsychiatric outcomes such as depression, anxiety, fatigue, and apathy are common post-stroke [25]. Depression post-stroke has an estimated pooled prevalence of around a third (27%) from observational studies. Those experiencing depression within 3 months post-stroke are at risk of remaining depressed a year after stroke [26]. Similarly, post-stroke anxiety can occur in 1 in 4 stroke-survivors [27]. Psychological outcomes such as depression and anxiety are often linked to cognitive deficits. Links between ethnicity and post-stroke outcomes such as depression are not consistent [28] with some finding no differences [29]. There do seem to be some ethnic differences when it comes to cognition with more cognitive impairment suggestive of dementia found in non-Europeans compared with Europeans [30]. Post stroke cognitive impairment is more common in those of Caribbean/African ethnicities [24]. In our study, at follow up, the use of psychological support was higher in Asian patients compared to White patients. Conversely, those from Mixed or Black ethnicities had similar levels to Whites in terms of receiving cognitive support. It is known that post-acute access to clinical psychology is often limited and below recommendations [31]. The degree of utilization of cognitive support offered to Black patients may be more of a reflection of the available clinical services or to cultural understanding of what normal or abnormal cognition may entail. Clinical services need to find ways to not only identify these individuals requiring psychological and/or cognitive support but also ways of following them up to ensure earlier detection of common post-stroke outcomes such as depression, anxiety, and cognitive impairment.

The paragraph beginning with Line 374 is confusing. Do the authors mean their study s representative of the nationally acquired data of SSNAP? The sentence in line 379 is unclear (The low 6-month follow-up data could be indicative of a poor reflection of the post-acute stroke population) and seems to be mean the patients are being blamed for non-attendance? I recommend this paragraph is rewritten for clarity.

**********

7. PLOS authors have the option to publish the peer review history of their article (what does this mean?). If published, this will include your full peer review and any attached files.

If you choose “no”, your identity will remain anonymous but your review may still be made public.

**Do you want your identity to be public for this peer review?**  For information about this choice, including consent withdrawal, please see our Privacy Policy.

Reviewer #1: **Yes:** Dennis Briley

**********

[NOTE: If reviewer comments were submitted as an attachment file, they will be attached to this email and accessible via the submission site. Please log into your account, locate the manuscript record, and check for the action link "View Attachments". If this link does not appear, there are no attachment files.]

To ensure your figures meet our technical requirements, please review our figure guidelines: https://journals.plos.org/plosone/s/figures

You may also use PLOS’s free figure tool, NAAS, to help you prepare publication quality figures: https://journals.plos.org/plosone/s/figures#loc-tools-for-figure-preparation.

NAAS will assess whether your figures meet our technical requirements by comparing each figure against our figure specifications.

# Author comment: Author response to Decision Letter 2

**DOI:** 10.1371/journal.pone.0354353.r004

2 Feb 2026

Please see attached response to reviewers document

Attachment Submitted filename: Response to reviewers.docx

# Peer review: Decision Letter 2

**DOI:** 10.1371/journal.pone.0354353.r005

**Authors:** Antony Bayer

15 Feb 2026

PONE-D-25-38926R2

Ethnic Disparities in the Longer-Term Clinical Care and Outcomes of Stroke Patients in England

PLOS One

Dear Dr. Tang,

Thank you for submitting your revised manuscript to PLOS ONE.

As you will see below, the reviewer remains concerned about the lack of provision of the data underlying Table 2 and (especially) Table 3, as previously highlighted. He also comments again about the problem of lumping together ischaemic and haemorrhagic stroke at 6 months follow up. Both issues would seem easily addressed by supplying additional data and I would ask you to consider this before any resubmission. I also noted a few typos consequent to your previous revision (e.g. line 62 "recurrence [5] IN non-White; lines 73 and 77 delete "excluding white minorities").

After further consideration, therefore, we feel your manuscript has merit but does not fully meet PLOS ONE’s publication criteria as it currently stands. Therefore, we invite you to submit a revised version of the manuscript that addresses the points raised.

Please submit your revised manuscript by Apr 01 2026 11:59PM. If you will need more time than this to complete your revisions, please reply to this message or contact the journal office at plosone@plos.org. When you're ready to submit your revision, log on to https://www.editorialmanager.com/pone/ and select the 'Submissions Needing Revision' folder to locate your manuscript file.

Please include the following items when submitting your revised manuscript:

- A letter that responds to each point raised by the academic editor and reviewer(s). You should upload this letter as a separate file labeled 'Response to Reviewers'.
- A marked-up copy of your manuscript that highlights changes made to the original version. You should upload this as a separate file labeled 'Revised Manuscript with Track Changes'.
- An unmarked version of your revised paper without tracked changes. You should upload this as a separate file labeled 'Manuscript'.

If you would like to make changes to your financial disclosure, please include your updated statement in your cover letter. Guidelines for resubmitting your figure files are available below the reviewer comments at the end of this letter.

If applicable, we recommend that you deposit your laboratory protocols in protocols.io to enhance the reproducibility of your results. Protocols.io assigns your protocol its own identifier (DOI) so that it can be cited independently in the future. For instructions see: https://journals.plos.org/plosone/s/submission-guidelines#loc-laboratory-protocols. Additionally, PLOS ONE offers an option for publishing peer-reviewed Lab Protocol articles, which describe protocols hosted on protocols.io. Read more information on sharing protocols at https://plos.org/protocols?utm_medium=editorial-email&utm_source=authorletters&utm_campaign=protocols.

We look forward to receiving your revised manuscript.

Kind regards,

Antony Bayer

Academic Editor

PLOS One

Journal Requirements:

If the reviewer comments include a recommendation to cite specific previously published works, please review and evaluate these publications to determine whether they are relevant and should be cited. There is no requirement to cite these works unless the editor has indicated otherwise.

[Note: HTML markup is below. Please do not edit.]

Reviewers' comments:

Reviewer's Responses to Questions

**Comments to the Author**

1. If the authors have adequately addressed your comments raised in a previous round of review and you feel that this manuscript is now acceptable for publication, you may indicate that here to bypass the “Comments to the Author” section, enter your conflict of interest statement in the “Confidential to Editor” section, and submit your "Accept" recommendation.

Reviewer #1: (No Response)

**********

2. Is the manuscript technically sound, and do the data support the conclusions?

The manuscript must describe a technically sound piece of scientific research with data that supports the conclusions. Experiments must have been conducted rigorously, with appropriate controls, replication, and sample sizes. The conclusions must be drawn appropriately based on the data presented.

Reviewer #1: Partly

**********

3. Has the statistical analysis been performed appropriately and rigorously?

Reviewer #1: I Don't Know

**********

4. Have the authors made all data underlying the findings in their manuscript fully available?

The PLOS Data policy requires authors to make all data underlying the findings described in their manuscript fully available without restriction, with rare exception (please refer to the Data Availability Statement in the manuscript PDF file). The data should be provided as part of the manuscript or its supporting information, or deposited to a public repository. For example, in addition to summary statistics, the data points behind means, medians and variance measures should be available. If there are restrictions on publicly sharing data—e.g. participant privacy or use of data from a third party—those must be specified.

Reviewer #1: No

**********

5. Is the manuscript presented in an intelligible fashion and written in standard English?

PLOS ONE does not copyedit accepted manuscripts, so the language in submitted articles must be clear, correct, and unambiguous. Any typographical or grammatical errors should be corrected at revision, so please note any specific errors here.

Reviewer #1: Yes

**********

6. Review Comments to the Author

Please use the space provided to explain your answers to the questions above. You may also include additional comments for the author, including concerns about dual publication, research ethics, or publication ethics. (Please upload your review as an attachment if it exceeds 20,000 characters)

Reviewer #1: My major concern continues to be the lack of supporting data for table 3. Table 3 purely shows the odds ratio. As a ridiculous example, it cannot be discerned if that means one white patient was taking an antiplatelet agent, one black person an antiplatelet agent for 173 days....based on the missing data listed in the supplementary tables for medication use, this would seem to be the logical conclusion. Table 1 which also presents odds ratio does provide the source data i.e. the absolute number of patients and percentages. With the large amount of missing data listed for medications listed in the supplementary tables on admission, the readers will need to know what degree of missingness the 6 month medication data is based on. The data for calculating the OR in table 2 should also be provided. This could be easily done by adding these data to supplementary table 2. I note that it is PLOS One policy that the data should be available: "The PLOS Data policy requires authors to make all data underlying the findings described in their manuscript fully available without restriction, with rare exception (please refer to the Data Availability Statement in the manuscript PDF file). The data should be provided as part of the manuscript or its supporting information, or deposited to a public repository. For example, in addition to summary statistics, the data points behind means, medians and variance measures should be available."

I again recommend that the data on medication use at the 6 month visit be purely for the ischaemic stroke patients. Historically, before imaging, stroke included both cerebral haemorrhage and ischaemic stroke, but they are not the same diseases and do not have the same need for medication, for example one would not treat a patient with cerebral haemorrhage with anticoagulation. To then conclude as the authors do in their abstract that “Black and Asian stroke-survivors experience worse outcomes and lower use of secondary prevention despite similar clinic attendance” when their data actually show different disease preponderance in different ethnic groups which are treated differently, is wrong. In my opinion the lumping together of ischaemic stroke and cerebral haemorrhage as one disease is not acceptable for this aspect of their work.

**********

7. PLOS authors have the option to publish the peer review history of their article (what does this mean?). If published, this will include your full peer review and any attached files.

If you choose “no”, your identity will remain anonymous but your review may still be made public.

**Do you want your identity to be public for this peer review?**  For information about this choice, including consent withdrawal, please see our Privacy Policy.

Reviewer #1: **Yes:** Dennis P. Briley

**********

[NOTE: If reviewer comments were submitted as an attachment file, they will be attached to this email and accessible via the submission site. Please log into your account, locate the manuscript record, and check for the action link "View Attachments". If this link does not appear, there are no attachment files.]

To ensure your figures meet our technical requirements, please review our figure guidelines: https://journals.plos.org/plosone/s/figures

You may also use PLOS’s free figure tool, NAAS, to help you prepare publication quality figures: https://journals.plos.org/plosone/s/figures#loc-tools-for-figure-preparation.

NAAS will assess whether your figures meet our technical requirements by comparing each figure against our figure specifications.

# Author comment: Author response to Decision Letter 3

**DOI:** 10.1371/journal.pone.0354353.r006

29 Jun 2026

Please see attached

Attachment Submitted filename: Response to reviewer.docx

# Editor report: Decision Letter 3

**DOI:** 10.1371/journal.pone.0354353.r007

**Authors:** Antony Bayer

8 Jul 2026

Ethnic Disparities in the Longer-Term Clinical Care and Outcomes of Stroke Patients in England

PONE-D-25-38926R3

Dear Dr. Tang,

We’re pleased to inform you that your manuscript has been judged scientifically suitable for publication and will be formally accepted for publication once it meets all outstanding technical requirements.

Within one week, you’ll receive an e-mail detailing the required amendments. When these have been addressed, you’ll receive a formal acceptance letter and your manuscript will be scheduled for publication.

An invoice will be generated when your article is formally accepted. Please note, if your institution has a publishing partnership with PLOS and your article meets the relevant criteria, all or part of your publication costs will be covered. Please make sure your user information is up-to-date by logging into Editorial Manager at Editorial Manager® and clicking the ‘Update My Information' link at the top of the page. For questions related to billing, please contact billing support.

If your institution or institutions have a press office, please notify them about your upcoming paper to help maximize its impact. If they’ll be preparing press materials, please inform our press team as soon as possible -- no later than 48 hours after receiving the formal acceptance. Your manuscript will remain under strict press embargo until 2 pm Eastern Time on the date of publication. For more information, please contact onepress@plos.org.

Kind regards,

Antony Bayer

Academic Editor

PLOS One

Additional Editor Comments (optional):

Reviewers' comments:

# Editor report: Acceptance letter

**DOI:** 10.1371/journal.pone.0354353.r008

**Authors:** Antony Bayer

PONE-D-25-38926R3

PLOS One

Dear Dr. Tang,

I'm pleased to inform you that your manuscript has been deemed suitable for publication in PLOS One. Congratulations! Your manuscript is now being handed over to our production team.

At this stage, our production department will prepare your paper for publication. This includes ensuring the following:

* All references, tables, and figures are properly cited

* All relevant supporting information is included in the manuscript submission,

* There are no issues that prevent the paper from being properly typeset

You will receive further instructions from the production team, including instructions on how to review your proof when it is ready. Please keep in mind that we are working through a large volume of accepted articles, so please give us a few days to review your paper and let you know the next and final steps.

Lastly, if your institution or institutions have a press office, please let them know about your upcoming paper now to help maximize its impact. If they'll be preparing press materials, please inform our press team within the next 48 hours. Your manuscript will remain under strict press embargo until 2 pm Eastern Time on the date of publication. For more information, please contact onepress@plos.org.

You will receive an invoice from PLOS for your publication fee after your manuscript has reached the completed accept phase. If you receive an email requesting payment before acceptance or for any other service, this may be a phishing scheme. Learn how to identify phishing emails and protect your accounts at https://explore.plos.org/phishing.

If we can help with anything else, please email us at customercare@plos.org.

Thank you for submitting your work to PLOS ONE and supporting open access.

Kind regards,

PLOS ONE Editorial Office Staff

on behalf of

Professor Antony Bayer

Academic Editor

PLOS One
